# A Systematic Review of Diffusion Models for Medical Image-Based Diagnosis: Methods, Taxonomies, Clinical Integration, Explainability, and Future Directions

**DOI:** 10.3390/diagnostics16020211

**Published:** 2026-01-09

**Authors:** Mohammad Azad, Nur Mohammad Fahad, Mohaimenul Azam Khan Raiaan, Tanvir Rahman Anik, Md Faraz Kabir Khan, Habib Mahamadou Kélé Toyé, Ghulam Muhammad

**Affiliations:** 1 Department of Computer Science, College of Computer and Information Sciences, Jouf University, Sakaka 72341, Saudi Arabia; 2 Department of Computer Science and Engineering, United International University, Dhaka 1212, Bangladesh; 3 School of Engineering and Energy, Murdoch University, Murdoch, WA 6150, Australia; 4 Department of Data Science and Artificial Intelligence, Monash University, Clayton, VIC 3153, Australia; 5 Department of Computer Science and Engineering, Ahsanullah University of Science and Technology, Dhaka 1208, Bangladesh; tranik.cse@gmail.com; 6 Department of Computer Science and Software Engineering, The University of Western Australia, Perth, WA 6009, Australia; 24427672@student.uwa.edu.au; 7 Department of Mathematics, Faculty of Science and Techniques, Dan Dicko Dankoulodo University of Maradi, Maradi 465, Niger; habib.kele@uddm.edu.ne; 8 Department of Computer Engineering, College of Computer and Information Sciences, King Saud University, Riyadh 11543, Saudi Arabia; ghulam@ksu.edu.sa

**Keywords:** diffusion models, medical imaging, artificial intelligence, explainable AI, clinician involvement

## Abstract

**Background and Objectives:** Diffusion models, as a recent advancement in generative modeling, have become central to high-resolution image synthesis and reconstruction. Their rapid progress has notably shaped computer vision and health informatics, particularly by enhancing medical imaging and diagnostic workflows. However, despite these developments, researchers continue to face challenges due to the absence of a structured and comprehensive discussion on the use of diffusion models within clinical imaging. **Methods:** This systematic review investigates the application of diffusion models in medical imaging for diagnostic purposes. It provides an integrated overview of their underlying principles, major application areas, and existing research limitations. The review followed the Preferred Reporting Items for Systematic Reviews and Meta-Analyses (PRISMA) 2020 guidelines and included peer-reviewed studies published between 2013 and 2024. Studies were eligible if they employed diffusion models for diagnostic tasks in medical imaging; non-medical studies and those not involving diffusion-based methods were excluded. Searches were conducted across major scientific databases prior to the review. Risk of bias was assessed based on methodological rigor and reporting quality. Given the heterogeneity of study designs, a narrative synthesis approach was used. **Results:** A total of 68 studies met the inclusion criteria, spanning multiple imaging modalities and falling into eight major application categories: anomaly detection, classification, denoising, generation, reconstruction, segmentation, super-resolution, and image-to-image translation. Explainable AI components were present in 22.06% of the studies, clinician engagement in 57.35%, and real-time implementation in 10.30%. Overall, the findings highlight the strong diagnostic potential of diffusion models but also emphasize the variability in reporting standards, methodological inconsistencies, and the limited validation in real-world clinical settings. **Conclusions:** Diffusion models offer significant promise for diagnostic imaging, yet their reliable clinical deployment requires advances in explainability, clinician integration, and real-time performance. This review identifies twelve key research directions that can guide future developments and support the translation of diffusion-based approaches into routine medical practice.

## 1. Introduction

Recent advancements in artificial intelligence (AI) have revolutionized various domains, and diffusion models have become one of the most promising innovations [1]. They have the capability of serving in numerous fields, such as computer vision [2,3,4,5,6], text generation, text sequence analysis [7,8,9,10,11], audio synthesis [8,12,13,14], and computational biology [15,16,17]. In recent years, diffusion models have seen rapidly growing use in computer vision, yielding strong results in segmentation, classification, generation, denoising, reconstruction, super-resolution, and image translation. They outperform traditional generative models, such as generative adversarial networks (GANs) [18], variational autoencoders (VAEs) [19], and transformers [20], achieving remarkable performance in different tasks. Diffusion models are increasingly important among generative models, and certain modifications enhance their effectiveness across domains and tasks. Their potential is especially notable in medical imaging, particularly image generation [21], segmentation [22], image translation [23], reconstruction [24], and many more.

Medical imaging has transformed in recent times, and traditional methods often struggle with noise and artifacts, especially in low-resolution 2D and 3D images from various modalities [25], including MRI, CT, ultrasound, and PET. This threatens the reliability of the diagnostic process. Recently, various diffusion models have been explored for real-world medical imaging tasks, demonstrating versatility and strong performance. They iteratively denoise and reconstruct images from noise, and their complex architectures capture meaningful patterns for anomaly detection and image classification. The most promising advantages are medical image synthesis and super-resolution, and their broad applicability consistently underscores their importance, with the growth in their use exponentially rising.

The research community has paid significant attention to implementing diffusion models. In addition, the diverse application of these models has resulted in a large number of research articles. This has deepened the struggle as well as the urgency to properly categorize these models based on their working mechanisms and functionality. Particularly in the medical domain, it is necessary to present a proper taxonomy and insights into these models according to the data modality and use cases. Prior studies [26,27,28,29] have discussed the foundations of diffusion models and categorized the models into different sections. A few studies focus specifically on the medical domain; however, they overlook key aspects, including clinician involvement. The previous surveys also did not address the explainability of diffusion models. Given the sensitivity of medical imaging, these issues are essential for assessing model reliability. In addition, data diversity is not clearly defined, and there is no thorough discussion of which diffusion methods are best suited to particular data types.

This study aims to address all these previous gaps in the existing studies. We present a holistic approach for the survey of diffusion models in medical imaging that incorporates the basic working mechanism of the diffusion model and in-depth literature analysis of diffusion model applications that have been exploited in medical imaging. A systematic taxonomy of the diffusion model is provided, highlighting the diffusion model in eight different tasks. Moreover, we present a comprehensive discussion section that provides insights and answers regarding the diffusion model. Finally, a future direction is proposed that provides guidelines and inspires the future research endeavors of this domain. The major contribution of this review work is as follows:

- The generic framework and mathematical operations of diffusion models are discussed for better comprehension.
- Five critical research questions are proposed to elucidate the key aspects of the diffusion model in the health informatics domain.
- Comprehensive literature analysis was performed to devise the categorization of the diffusion model for the delegated domain.
- We did not restrict the review work to a single image modality; we instead covered all widely utilized image modalities, aiming to improve the diversification of the work.
- Finally, future recommendations are given to guide future researchers and improve the efficiency of their work.

**Figure 1:**
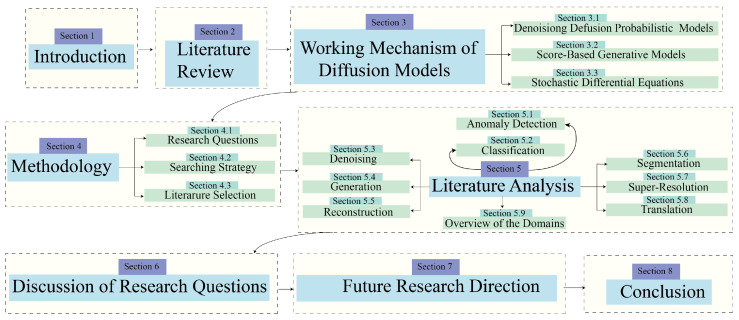
Workflow of the proposed review process.

Figure 1 outlines the sequence of actions in the proposed review process.

## 2. Related Works

This section summarizes the existing studies that review the foundation of diffusion models and their application in different domains. Additionally, a gap analysis was performed that clarifies the motivations of this proposed research.

Reference [26] provided a comprehensive review of diffusion models, particularly applied in the computer vision field. It presented a generic diffusion model framework and discussed its relationship with the generative models. And finally, the authors reviewed several applications of different models in various computer vision tasks such as image reconstruction, segmentation, and translation. Another study [27] also served the same purpose; it summarized different types of diffusion models and their connection with other generative models and finally concluded by discussing the application of the diffusion model. In addition, the authors also discussed the sampling technique. However, neither study focused on the specific application of the diffusion model but rather provided the foundational knowledge of the diffusion model across various domains. Reference [30] conducted a survey on generative diffusion models. It emphasized the background information and algorithmic development of generative models, including diffusion models. Reference [29] presents a comprehensive review of diffusion models in medical imaging. The authors taxonomized the diffusion models based on the algorithm. Moreover, the study discussed the potential use cases of these models in the medical domain. Reference [28] also focused on medical imaging and explicitly highlighted the emerging applications of diffusion models in MRI. It categorized models based on their applications, such as segmentation, anomaly detection, and translation. Additionally, it provided adequate background theory of diffusion models. Despite brief discussions, it narrowed down its review scope as it emphasized only a single image modality. Reference [31] focused on lightweight diffusion models. It discussed architectural quantization, network quantization, and architecture pruning of the model. Finally, it described the utilization of diffusion models in the field of image classification. Reference [32] provided a coherent review of diffusion models in multimodal data. It specifically highlighted controllable diffusion generation. Moreover, it investigated the performance metrics utilized for the performance assessment of the diffusion model. However, the foundational knowledge of diffusion methods was not properly explained in the study.

Along with the computer vision field, diffusion models are employed in bioinformatics and computational biology. Reference [15] investigated the theoretical foundations of diffusion models and explored their applications in bioinformatics. Moreover, it highlighted open-source tools and a repository of diffusion models for the future applications of this domain. Reference [33] proposed a new generative framework to demonstrate the utilization of diffusion models in several seismic tasks. It particularly described demultiplex, denoising, and interpolation case studies. The evaluation of the previous work effectively contributes to the geoscience community. In the next study [34], the authors presented a comprehensive review of the advances of generative AI, including GANs, GPTs, diffusion models, and transformers. They encompassed the application of these models in different domains, including healthcare, education, business, and entertainment. Diffusion models also have extensive advantages in synthesizing time series data. The authors of [35] investigated diffusion models for time-series forecasting, imputation, and generation. They described the functionality of several widely used time series diffusion methods and provided a brief comparison. However, no proper taxonomy or categorization of diffusion models in the time-series domain is present. Reference [36] presented a survey to explore the interplay between the representation learning and diffusion models. It discussed the basic mathematical expressions and architectures of these models and finally presented a taxonomy between the representation learning and diffusion models, including the key area of concern and potential exploration. Nevertheless, it did not discuss adequate applications of the diffusion models. Image generation and editing using the diffusion model have already gained ample interest due to their diverse utilization. The authors of [37] investigated the existing diffusion methods utilized for image editing, encompassing their theoretical and practical description. They show the performance evaluation of these benchmark models. Finally, they provide a categorization based on their functionality. Though they reviewed the image editing tasks using diffusion models, many other scopes, including image generation, segmentation, translation, and reconstruction, were not properly addressed.

Despite significant attention being paid to the surveying of diffusion-based methods, adequate research has not been conducted from the perspective of medical imaging. This is also a key area of computer vision. Most studies did not highlight the significant advantages of diffusion models in health informatics, instead focusing on multiple domains; therefore, only ground-level knowledge can be obtained. On the contrary, some studies focus on specific applications or on specific image modalities. Consequently, they did not emphasize dataset diversification. Diffusion models have the unique advantage of creating synthetic images or reconstructing them. They require a proper clinician to properly annotate the synthetic images, but only one study underscores this clinician’s importance. In addition, the explainability of these diffusion models was not properly investigated, which could increase the reliability. This study aims to address all these challenges systematically, providing a comprehensive review of diffusion methods in the field of medical imaging. Table 1 includes the comparison of this proposed research work with the existing work.

## 3. Working Mechanism of Diffusion Models

**Figure 2:**
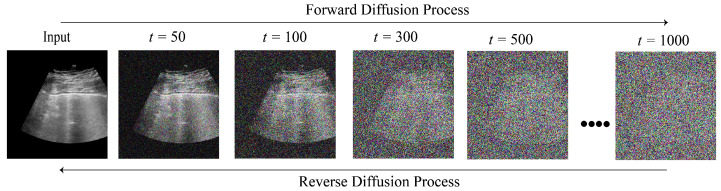
Visualization of the diffusion process applied to an ultrasound image, where the forward process incrementally adds Gaussian noise over time steps (*t* = 50) to *t* = 1000, progressively degrading the image to pure noise, while the reverse process attempts to reconstruct the original image by iteratively denoising it from the final noisy representation.

Diffusion models are advanced probabilistic generative models that progressively degrade data over several steps by injecting noises and then learn to reverse the diffusion process to synthesize noise-free data [26]. By simply denoising a random initial sample of pure noise, it is possible to generate new data points that resemble the training data from a trained diffusion model [38]. Recently, these models have capitalized on the advanced application of computer vision, especially in the medical domain and have become widely employed for medical image reconstruction, super-resolution, translation, etc. These models comprise two processes: forward and reverse. The forward process gradually distorts the input data by adding noise, whereas the reverse process removes the noises and recreates the original images [39]. Figure 2 illustrates the forward and reverse diffusion of an ultrasound image at different time steps. Although diffusion models have been developed in various forms, three fundamental diffusion models serve as the foundation for these advancements: Denoising diffusion probabilistic models (DDPMs) [3,40], score-based generative models (SGMs) [41], and stochastic differential Equations (SDEs) [42,43]. A comprehensive comparison of these techniques is discussed in the following section.

### 3.1. Denoising Diffusion Probabilistic Models (DDPMs)

A denoising diffusion probabilistic model (DDPM) employs two Markov chains: a forward chain in which Gaussian noise perturbs data in successive steps and a reverse chain to obtain noise-free synthetic data [44].

Forward Process: The forward process initiates with the addition of Gaussian noise. Assume $p(x_{0})$ is the data density of the uncorrupted image. Given a training sample distribution of $x_{0}∼q\left(x_{0}\right)$, and the corresponding noisy images are $\left(x_{1},x_{2},\ldots ,x_{T}\right)$, which are obtained by the following sequential Markovian process:

(1) $$q\left(x_{t}|x_{t-1}\right)=N\left(x_{t};\sqrt{1-\beta_{t}}\cdot x_{t-1},\beta_{t}\cdot I\right),\;\forall t\in \left\{1,\ldots ,T\right\}$$

where *T* and $\beta_{1},\ldots ,\beta_{T}\in \left(0,1\right)$ refer to the number of diffusion steps and the variance schedule across diffusion steps, respectively, and I is the identity matrix. The normal distribution evaluated at *x* with mean μ and covariance Σ is denoted by N(x;μ,Σ). The sample $x_{t}$ is obtained by directly sampling from this distribution, where the timestamp *t* is selected uniformly from {1,…,T}.

(2) $$q\left(x_{t}|x_{0}\right)=N\left(x_{t};\sqrt{{\bar{\alpha }}_{t}}x_{0},\left(1-{\bar{\alpha }}_{t}\right)I\right)$$

(3) $$x_{t}=\sqrt{{\bar{\alpha }}_{t}}x_{0}+\sqrt{1-{\bar{\alpha }}_{t}}\epsilon$$

where ϵ∼N(0,I). Using $\alpha_{t}=1-\beta_{t}$ and ${\bar{\alpha }}_{t}=\prod_{s=1}^{t}\alpha_{s}$, we can obtain a noisy version $x_{t}$ through a single step from the original image $x_{0}$ and establish a fixed variance schedule $\beta_{t}$.

Reverse Process: As T→∞, the latent $x_{T}$ converges to an isotropic Gaussian distribution, which refers to a multivariate distribution where each dimension has equal variance and is independent of the others. In learning the reverse distribution of $p_{\theta }\left(x_{t-1}|x_{t}\right)$, we sample $x_{T}$ from a normal distribution, N(0,I), and finally acquire a new data point. To estimate $p_{\theta }\left(x_{t-1}|x_{t}\right)$, we can parameterize the process using the following equations:

(4) $$p_{\theta }\left(x_{0:T}\right)=p\left(x_{T}\right)\prod_{t=1}^{T}p_{\theta }\left(x_{t-1}|x_{t}\right)$$

(5) $$p_{\theta }\left(x_{t-1}|x_{t}\right)=N\left(x_{t-1};\mu_{\theta }\left(x_{t},t\right),\Sigma_{\theta }\left(x_{t},t\right)\right)$$

Here, $\mu_{\theta }\left(x_{t},t\right)$ and $\Sigma_{\theta }\left(x_{t},t\right)$ are the mean and variance, respectively. This process is sampled iteratively until t=1 and achieves the generated result ${\hat{x}}_{0}∼p\left(x_{0}\right)$.

To train the model and ensure that $p(x_{0})$ accurately learns the original data distribution $q(x_{0})$, we could optimize the variational bound on negative log-likelihood:

(6) $$\begin{array}{cc} E[-\log p_{\theta }\left(x_{0}\right)] & \leq E_{q}\left[-\log\frac{p_{\theta }\left(x_{0:T}\right)}{q(x_{1:T}|x_{0})}\right]\\ \; & =E_{q}\left[-\log p\left(x_{T}\right)-\sum_{t=1}^{T}\log\frac{p_{\theta }\left(x_{t-1}|x_{t}\right)}{q(x_{t}|x_{t-1})}\right]\\ \; & =:L \end{array}$$

In this training period, the main objective of the neural network is to generate as close as possible to the true posterior of the forward process in each time step, *t*, by estimating the noise addition. Ho et al. [3] proposed a novel approach that simplified the training session by modifying the mean, $\mu_{\theta }$.

### 3.2. Score-Based Generative Models (SGMs)

The fundamental concept of score-based generative models is to distort data quality by introducing a series of increasing Gaussian noise and training deep neural network models to estimate the score functions for noisy data distributions simultaneously. This technique is also recognized as a noise conditional score network (NCSN) [41]. This method involves generating samples by sequentially applying the score functions at decreasing levels of noise using several score-based sampling approaches, including Langevin Monte Carlo [45], stochastic differential Equations [46], ordinary differential Equations [47], and their various combinations. In the development of score-based generative models, the processes of training and sampling are completely distinct, meaning that after estimating the score functions, a wide range of sampling approaches can be employed. For better comprehension, let us assume $q(x_{0})$ is the data distribution, and $0<\sigma_{1}<\sigma_{2}<\ldots <\sigma_{t}<\ldots <\sigma_{T}$ is a sequence of noise levels. A generic example of SGMs involves distorting a data point $x_{0}$ to $x_{t}$ by the Gaussian noise distribution $q\left(x_{t}|x_{0}\right)=N\left(x_{t};x_{0},\sigma_{t}^{2}I\right)$. This results in a sequence of noisy data densities $q\left(x_{1}\right),q\left(x_{2}\right),\ldots ,q\left(x_{t}\right)$ where $q\left(x_{t}\right)=\int q\left(x_{t}\right)q\left(x_{0}\right)dx_{0}$.

(7) $$x_{t}=x_{t-1}+\sqrt{\sigma_{t}^{2}-\sigma_{t-1}^{2}}z_{t},\;z_{t}∼N\left(0,I\right)$$

(8) $$x_{t-1}=x_{t}+\left(\sigma_{t}^{2}-\sigma_{t-1}^{2}\right)\nabla_{x_{t}}\log q\left(x_{t}\right)+\left(\sqrt{\sigma_{t}^{2}-\sigma_{t-1}^{2}}\right)z_{t}$$

The forward and reverse diffusion processes of SGMs are expressed by Equations (7) and (8).

A noise-conditional scoring network refers to a deep neural network, denoted as $s_{\theta }$, that is trained for estimating the score function $\nabla_{x_{t}}\log q\left(x_{t}\right)$. Several learning score functions are utilized, such as score matching [48], denoising score matching [49,50], and sliced score matching [51]. One of them can be employed to train the noise conditional networks, and the following equations indicate the modification when they are added to Equation (9).

(9) $$\begin{array}{cc} \; & E_{t∼U\left[1,T\right],x_{0}∼q\left(x_{0}\right),x_{t}∼q\left(x_{t}|x_{0}\right)}\left[\lambda \left(t\right)\sigma_{t}^{2}{∥\nabla_{x_{t}}\log q\left(x_{t}\right)-s_{\theta }\left(x_{t},t\right)∥}^{2}\right]\\ \; & \overset{\left(i\right)}{=}E_{t∼U\left[1,T\right],x_{0}∼q\left(x_{0}\right),x_{t}∼q\left(x_{t}|x_{0}\right)}\left[\lambda \left(t\right)\sigma_{t}^{2}{∥\nabla_{x_{t}}\log q\left(x_{t}|x_{0}\right)-s_{\theta }\left(x_{t},t\right)∥}^{2}\right]+\mathrm{const}\\ \; & \overset{\left(ii\right)}{=}E_{t∼U\left[1,T\right],x_{0}∼q\left(x_{0}\right),x_{t}∼q\left(x_{t}|x_{0}\right)}\left[\lambda \left(t\right){∥-\frac{x_{t}-x_{0}}{\sigma_{t}}-\sigma_{t}s_{\theta }\left(x_{t},t\right)∥}^{2}\right]+\mathrm{const}\\ \; & \overset{\left(iii\right)}{=}E_{t∼U\left[1,T\right],x_{0}∼q\left(x_{0}\right),\epsilon ∼N\left(0,I\right)}\left[\lambda \left(t\right)∥\epsilon +\sigma_{t}s_{\theta }\left(x_{t},t\right){∥}^{2}\right]+\mathrm{const} \end{array}$$

All these equations are ultimately the result of modification with Equation (9); hence, it is justified that the training objectives of both DDPMs and SGMs are equivalent. To generate samples, SGMs leverage iterative approaches that produce samples from $s_{\theta }\left(x,T\right),s_{\theta }\left(x,T-1\right),s_{\theta }\left(x,T-2\right),\ldots ,s_{\theta }\left(x,0\right)$ in succession. Several sampling methods exist due to the decoupling of training. One of the techniques is called annealed Langevin dynamics (ALD) [41]. In this section, *N* is the iteration number per time step, and $s_{t}>0$ is the step size. ALD is declared with $x_{T+1}^{\left(N\right)}∼N\left(0,I\right)$, then Langevin Monte Carlo is applied for t=T,T−1,…,1. At each time step 0≤t<T, the process starts with $x_{t}^{\left(0\right)}=x_{t+1}^{\left(N\right)}$ according to the updated rule for i=0,1,…,N−1:

(10) $$\begin{array}{cc} \epsilon^{\left(i\right)} & \leftarrow N(0,I)\\ x_{t}^{\left(i+1\right)} & \leftarrow x_{t}^{\left(i\right)}+\frac{1}{2}s_{t}s_{\theta }\left(x_{t}^{\left(i\right)},t\right)+\sqrt{s_{t}}\epsilon^{\left(i\right)} \end{array}$$

The theory of Langevin Monte Carlo [52] guarantees that as $s_{t}\to 0$ and N→∞, $x_{0}^{\left(N\right)}$ is a valid sample from the data distribution $q(x_{0})$.

### 3.3. Stochastic Differential Equations (SDEs)

The concept of perturbing data in a typical diffusion process is extended to an infinite number of noise scales using continuous-time differential equations, known as stochastic differential Equations (SDEs). Like the aforementioned methods, this technique also gradually converts the data distribution $p(x_{0})$ into noise. However, this is accomplished with an SDE. As demonstrated in Reference [53], the reverse diffusion process can be represented by a reverse time SDE. This SDE implies the score function at each time step. The following equation denotes the forward diffusion process of an SDE:

(11) dx=f(x,t)dt+σ(t)dω

Here, ω is the standard Wiener process, *f* is the function, *x* and *t* are the coefficients, and σ is the diffusion coefficient which controls the addition of noise.

(12) $$dx=\left[f\left(x,t\right)-\sigma {\left(t\right)}^{2}\nabla_{x}\log p_{t}\left(x\right)\right]dt+\sigma \left(t\right)d\hat{\omega }$$

Equation (12) refers to the reverse diffusion process of an SDE. To obtain the noise-free images, $\sigma {\left(t\right)}^{2}\nabla_{x}\log p_{t}\left(x\right)$ is subtracted. In the next stage, the neural network model is trained using the following training objective:

(13) $$\arg\min_{\theta }E_{t}\left[\lambda \left(t\right)E_{p(x_{0})}E_{p_{t}\left(x_{t}|x_{0}\right)}\left[∥s_{\theta }\left(x_{t},t\right)-\nabla_{x_{t}}\log p_{t}\left(x_{t}|x_{0}\right){∥}_{2}^{2}\right]\right]$$

A brief comparison of these techniques is demonstrated in Table 2 for effective comprehension. Recent advances have extended the role of diffusion models beyond conventional image generation and reconstruction, positioning their versatility within medical imaging in AI pipelines. In addition to their capacity to model complex data distributions, diffusion processes can be systematically utilized to analyze and interpret the behavior of diagnostic algorithms. Gradient-based [54,55] and attention-guided [56,57] diffusion frameworks have been proposed to monitor the evolution of internal feature representations along both the forward and reverse diffusion trajectories, clarifying which anatomical regions most strongly shape the model outputs. In parallel, counterfactual image synthesis methods, such as AnoDDPM [51] and DreaMR [58], exploit diffusion-based priors to generate realistic synthetic medical images while making only minimal changes to disease-related findings. These counterfactuals allow clinicians and researchers to study how small, controlled image variations influence downstream model decisions, thereby linking generative modeling with interpretable AI.

In the aforementioned section, we primarily focus on the standard DDPM and score-based diffusion models. Although these approaches form the core mathematical understanding of diffusion models, their practical use in medical imaging has increasingly moved toward more task-specific backbone architectures. A key advancement is the development of diffusion bridge models, such as I2SB [59] and SelfRDB [60], which rethink noise injection and the forward process. Rather than relying on Gaussian noise as the main perturbation, these models learn mappings between raw and fully reconstructed images distributions using diffusion-like stochastic and deterministic formulas. This framework directly incorporates task-specific information and maintains structural dependencies present in medical images. Consequently, these methods achieve improved reconstruction quality, faster training, and greater alignment with real-world medical imaging workflows.

In parallel, diffusion-based methods incorporate architectural refinements that explore new backbones capable of capturing long-range dependencies and high-resolution medical structures. The performance of these hybrid models is significantly promising, prompting robust model development in this domain. Traditional U-Net and transformer designs are increasingly augmented by state-space backbones such as Mamba [61]. Early diffusion frameworks using Mamba-style architectures such as I2I-Mamba [62] and VM-DDPM [63] show advantages in selective state-space recurrence, memory efficiency, and global context modeling. Hybrid generative frameworks that combine diffusion models with scalable autoregressive components are emerging as especially promising. Methods like MambaRoll [64] and VAR [65] aim to pair the high-fidelity sampling of diffusion models with the sequential consistency and scalability of autoregressive approaches, attaining higher sample quality and stronger modeling of long-range structural coherence. These hybrid architectures that merge in diffusion pipelines significantly boost the overall performance. Overall this section provides an understanding of the widely used diffusion models’ working mechanisms and the recent evolution of development of this technique in medical imaging, which together provide a comprehensive overview of its strength and relevance.

## 4. Methodology

This study adopted a systematic approach to conduct the review work efficiently. This section includes our literature searching and selection strategy and how the literature was organized into several domains for further analysis.

### 4.1. Research Questions (RQs)

This study formulated well-defined research questions (Table 3) to state the objectives and ensure relevance to our respective fields. The research questions were derived through a structured review process to narrow down our research focus. We identified five key sections—model performance, dataset heterogeneity, explainability, clinical applicability, and future research directions—and mapped each question to ensure comprehensive coverage of the methodological aspects of diffusion-based frameworks. The questions were explicitly designed to address the gaps observed in existing review works. In addition, they provide a clear motivation for this study at a glance.

### 4.2. Searching Strategy

Searching for relevant articles is essential to conducting a comprehensive review. We utilized three scientific databases and repositories for the search process: Scopus, Web of Science, and Google Scholar. Customized search queries were used to optimize the literature searching process and focus only on the health informatics articles that exploit diffusion methods. The queries utilized in this study are listed in Table 4. From this table, it is observed that the diffusion models or methods are the primary queries, different techniques of medical imaging are regarded as secondary query, and, finally, the connection between these queries results in the relevant articles for the review work.

**Figure 3:**
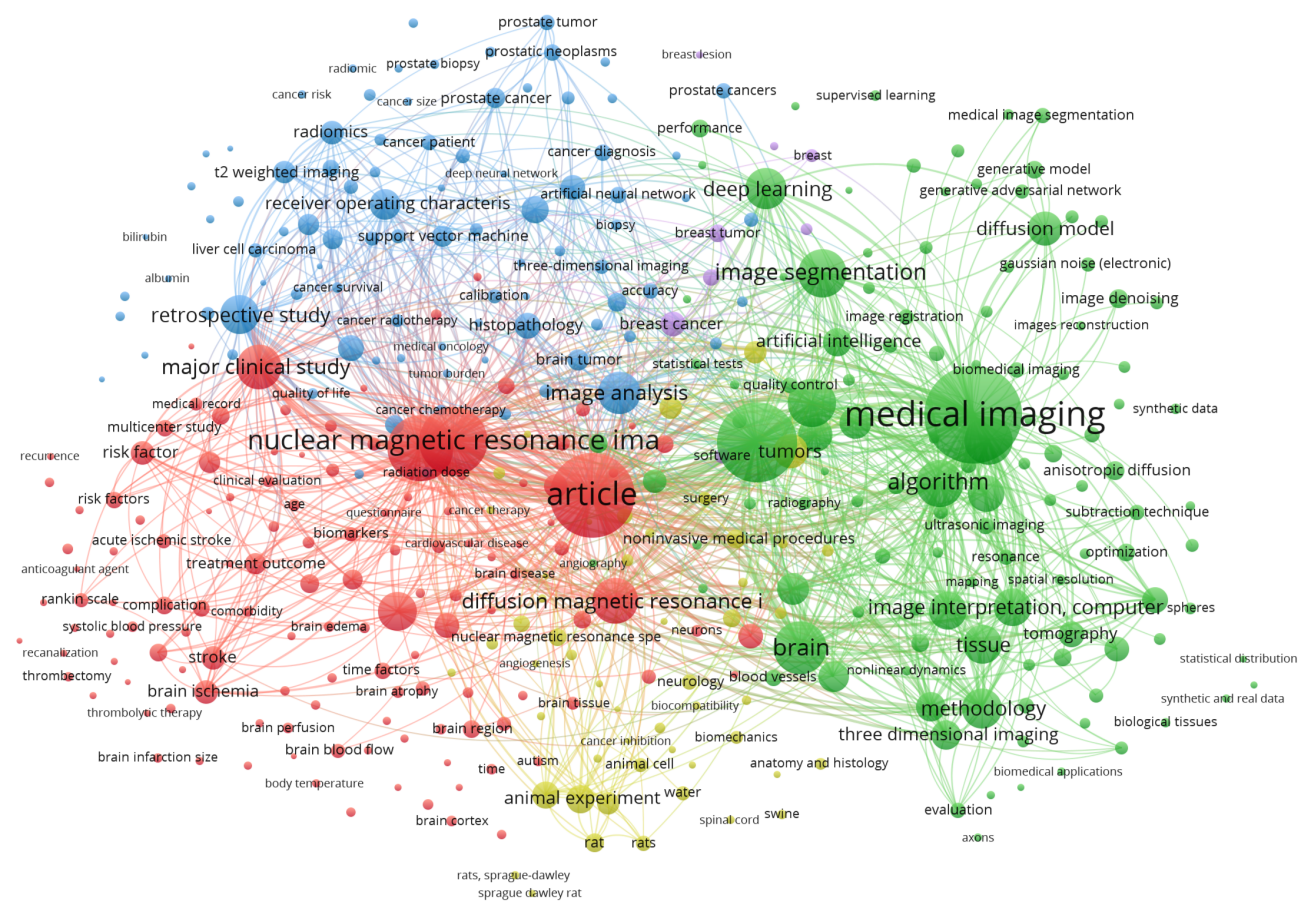
Bibliometric network visualization illustrating the co-occurrence of key terms in the research literature related to medical imaging and diffusion models, with clustered nodes representing thematic research areas.

Additionally, we created a bibliometric network using the VOSviewer (https://www.vosviewer.com/, accessed on 12 December 2025). These queries are mainly the index keywords that result in a broad range of academic articles. To effectively represent these keywords, we visualize them in Figure 3. This figure emphasizes several keywords that are highly associated with diffusion models and medical imaging.

### 4.3. Literature Selection

**Figure 4:**
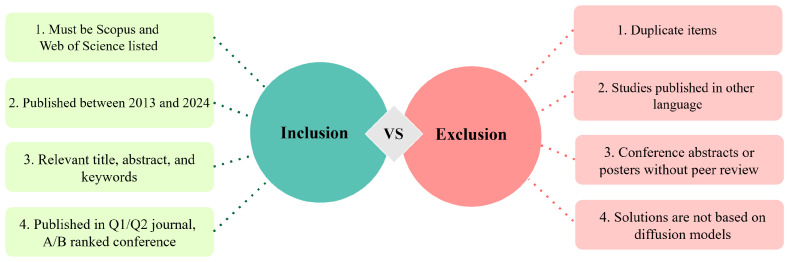
Paper inclusion and exclusion criteria of the review, presenting a structured selection methodology by outlining inclusion criteria to identify relevant papers while specifying exclusion criteria to filter out irrelevant papers.

In accordance with the systematic literature analysis, we established certain criteria for inclusion (IC) and exclusion (EC) when selecting the final research papers to extract essential information regarding the application of the diffusion model in health informatics. Figure 4 displays the proposed inclusion and exclusion criteria of this review work. It also provides transparency and indicates a systematic approach to paper selection.

**Figure 5:**
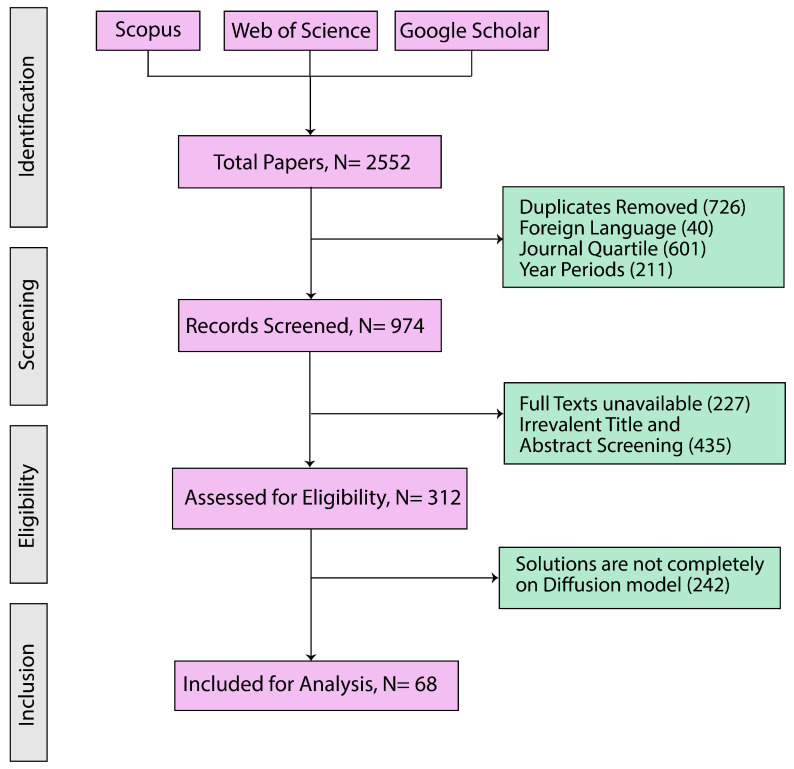
The systematic paper selection outlining the progressive filtering of studies through four stages: identification (2552 records), screening (974 records), eligibility assessment (312 records), and final inclusion (68 studies).

Figure 5 illustrates the step-by-step process of selecting research papers and summarizes the Preferred Reporting Items for Systematic Reviews and Meta-Analyses (PRISMA) diagram, which outlines the systematic review process employed in this study [66]. We have followed the PRISMA 2020 statement, an updated guideline for reporting systematic reviews [67], and all materials are provided in the Supplementary Files. In the initial identification phase, 2552 papers published between 2013 and 2024 were identified based on the search strategies described in Table 4. Only papers available in both the Scopus and Web of Science databases were considered, and duplicate entries were excluded. This stage aligned with Inclusion Criteria 1 and 2, as well as Exclusion Criterion 1. During the subsequent screening phase, the titles and abstracts of these papers were reviewed. Only studies published in English were retained, in accordance with Inclusion Criterion 3 and Exclusion Criterion 2. This process resulted in a refined pool of 974 papers. In the eligibility phase, a more rigorous evaluation was conducted based on Inclusion Criterion 4: the papers had to be published in Q1 or Q2 journals according to the SCImago Journal Rank. Conference abstracts or posters without peer review were excluded, following Exclusion Criterion 3. This led to the selection of 312 eligible papers. Throughout the review work, we prioritized Q1/Q2 journals or A/B-ranked conferences to ensure strong methodological and peer-review standards. These platforms ensure stricter evaluations, improving consistency and reliability. While this may exclude some recent work from lower-ranked platforms, we prioritize high-quality, well-validated, and widely recognized diffusion-model studies in health informatics to provide scientifically reliable research. In between the eligibility and last inclusion phase, a full-text review was conducted. Papers that did not present solutions based on diffusion models in imaging were excluded, in line with Exclusion Criterion 4. Finally, in the inclusion phase, 68 articles met all inclusion and exclusion criteria and were included in our final analysis.

**Figure 6:**
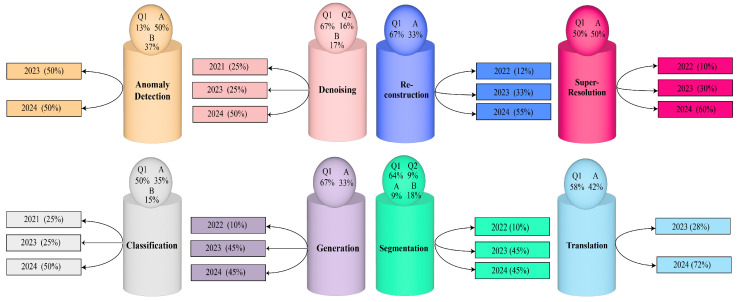
Distribution of the selected studies across various research domains in health informatics, categorized by publication types—Q1 and Q2 journals and A/B-ranked conferences—highlighting that domains such as image denoising, reconstruction, and generation have a higher concentration of Q1 publications, while anomaly detection and image classification exhibit a more balanced representation across journals and conferences.

To facilitate understanding, these papers were categorized into 8 different domains, namely, (1) anomaly detection, (2) image classification, (3) image denoising, (4) image generation, (5) image segmentation, (6) image reconstruction, (7) image super-resolution, and finally (8) image translation. An overview of the categorization of these selected articles is illustrated in Figure 6. Additionally, the quality and the published years of the journals are mentioned to maintain the fairness of the review work.

**Figure 7:**
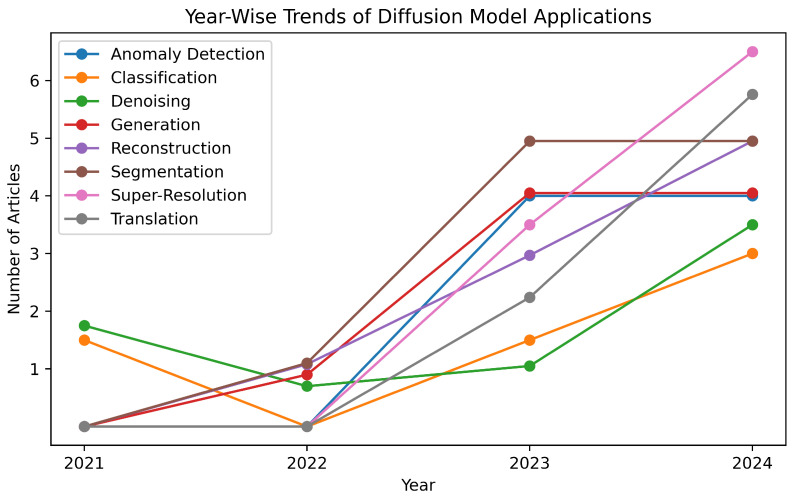
Graphical representations of diffusion model publication trends over years.

Figure 7 depicts the year-wise progression of diffusion-model applications across all the eight medical imaging domains. The trend indicates a marked increase since 2022 in most cases, including classification, segmentation, super-resolution, and translation, reflecting growing methodological and task-specific adoption.

## 5. Literature Analysis

In this section, we review studies that leveraged diffusion-based models across eight different medical imaging domains, namely, anomaly detection, classification, denoising, generation, reconstruction, segmentation, super-resolution, and translation. These studies are evaluated based on different imaging modalities, model performance, XAI techniques, clinician involvement, and real-time implementation, which identifies key advancements as well as gaps that highlight areas for future research.

### 5.1. Anomaly Detection

Anomaly detection holds significant importance within health informatics [68] as it facilitates early diagnosis and treatment by identifying aberrations or deviations from established normal patterns [69]. In this regard, diffusion models emerge as highly promising due to their advanced generative capacities [51,70,71,72]. Specifically, within the domain of health informatics, diffusion models exhibit the capability to accurately detect subtle anomalies residing within intricate medical data, encompassing MRI scans, CT images, and dermatological assessments. By employing these models, anomaly detection accuracy can be heightened, instances of false positives can be minimized, and a more profound comprehension of anomalous patterns can be attained.

The reviewed studies demonstrate the versatility of diffusion models in various health informatics tasks, particularly in medical imaging, as shown in Table 5. References [70,73] achieved high levels of specificity and accuracy in brain tumor segmentation and image restoration tasks using conditional-guided and conditioned diffusion models, respectively. In contrast, References [71,74] focused on different modalities, such as CT scans and dermatological images, achieving noteworthy accuracy and ROC scores, although some challenges were observed in segmentation performance. Other studies, such as [51,72], employed multi-scale noise diffusion and patched diffusion models but encountered variability in results depending on the dataset, highlighting the importance of selecting appropriate models based on the specific tasks and data types. The inclusion of explainability features varied among the studies, with some, like [75], emphasizing interpretability, while others did not focus on this aspect.

In the case of RQ1, it is evident that regarding the effectiveness of diffusion models across anomaly detection in health informatics, models like conditional-guided diffusion and conditioned diffusion have shown high specificity and accuracy, particularly in MRI-based anomaly detection and image restoration tasks. These models excel in segmentation tasks, identifying subtle irregularities with precision. For RQ2, regarding the diversity and comprehensiveness of datasets, the predominant use of MRI datasets—constituting about 62.5%—alongside CT and dermatological images suggests that diffusion models are being tested across a broad spectrum of imaging modalities. However, there is still room for more diversity in data types. Regarding explainability, as mentioned in RQ3, roughly half of the models incorporate explainability features. For instance, models that include interpretability tools or enhanced transparency mechanisms such as confidence maps or pixel based anomaly maps are more readily accepted in clinical environments, aiding clinicians in understanding and trusting the model outputs. As for clinician involvement denoted in RQ4, about 62.5% of the studies show that these models are developed with direct input from healthcare professionals, ensuring that the models are not only technically sound but also clinically relevant. However, a significant gap remains in real-time deployment in RQ5 as none of the diffusion models reviewed have been successfully integrated into live clinical workflows. This emphasizes the need for further research and technological advancements to overcome real-time applications’ latency and computational challenges.

### 5.2. Classification

Classification tasks in health informatics contribute to the diagnosis of diseases, patient stratification, and treatment decision-making [77]. Diffusion models, known for their sophisticated generative capabilities, provide substantial advantages by efficiently capturing intricate patterns from various medical data sources, including X-rays, retinal OCTs, and histopathology images [77,78]. These models are crucial in scenarios where conventional classification algorithms face challenges due to variability and noise in medical imaging, thereby enabling more precise and robust predictions. The capacity of diffusion models to refine and enhance features before classification holds the potential to enhance diagnostic accuracy and facilitate personalized healthcare.

The reviewed studies illustrate diffusion models’ broad application and effectiveness in various medical classification tasks. As Table 6 shows, References [77,79] utilized Latent Diffusion and dual-granularity conditional guidance diffusion models, achieving high accuracy in tasks such as cataract detection, diabetes recognition, and dermatological assessments. These models achieved accuracy rates exceeding 90% in several cases. The studies also emphasize the robustness of diffusion models across multiple imaging modalities, including X-rays, retinal OCTs, and ultrasound images. In contrast, References [78,80] examined models like the Semantic Diffusion and Synchronized Anisotropic Diffusion Equation, respectively. These models demonstrated varying degrees of success depending on the complexity of the dataset. The former achieved moderate accuracy in histopathology images, while the latter excelled in MRI and CT scan analysis. Furthermore, References [81,82] contributed to the field by focusing on dermatology and breast cancer datasets. They applied Accuracy Adaptive Sampling and distance-aware diffusion models, respectively, highlighting the significance of model selection tailored to the data type. From Table 6, it is also observed that the diffusion-based framework obtains consistently high accuracies of braille classification of 93.50%, diabetic retinopathy of 93.75%, and ultrasound-based PMG2000 93.1% on binary classification dataset. Performance drops noticeably on more challenging datasets such as on GLySAC 71.6%, and APTOS2019 of 85.8%. Overall, this analysis showed that the performance of diffusion models can significantly vary based on the dataset and specific classification task.

RQ1 pertains to the effectiveness of diffusion models in health informatics classification tasks. The results indicate high accuracy across various conditions, such as cataract detection and dermatological classifications when utilizing models like Latent Diffusion and dual-granularity conditional guidance. RQ2 focuses on the diversity of datasets used in these studies. These datasets encompass multiple imaging modalities, including X-rays, retinal OCTs, and histopathology. The majority of the datasets are public, although the comprehensiveness is limited due to the lack of diverse pathologies. RQ3 addresses the importance of explainability. It is worth noting that none of the reviewed studies explicitly incorporate explainability features except [82], where the authors utilized signed distance maps (SDMs) for calculating pixel distance compared to the target label, enhancing transparency with model prediction. Furthermore, clinician involvement is minimal, with no reported direct involvement in these studies. This suggests a potential disconnect between model development and clinical application, an area of concern for our RQ4. Lastly, the implementation of real-time deployment remains a challenge that has yet to be fully addressed in any of the studies. This indicates the need for further advancements to reduce computational complexity and latency, making this approach more practical for clinical use. This issue is the primary focus of RQ5.

### 5.3. Denoising

Denoising is an essential process in health informatics, specifically in medical imaging as noise may significantly reduce the accuracy of diagnoses [22]. Diffusion models are becoming more commonly used for denoising applications due to their ability to effectively restore clean images from noisy input while maintaining crucial structural information [83,84,85]. This ability is essential for improving the quality of images obtained from modalities such as MRI, PET, and X-rays, resulting in more accurate clinical interpretations and improved patient outcomes.

Using sophisticated denoising techniques provided by diffusion models enhances image clarity and diagnostic precision, crucial in intricate medical evaluations. The reviewed research utilizes diverse diffusion models to perform denoising tasks on distinct imaging modalities, illustrated in Table 7. References [83,86] utilized feature map denoising diffusion and Generalized Hybrid Denoising Diffusion models on various datasets, including ISIC, Kvasir, BUSI, and chest X-ray. Their experiments demonstrated excellent performance, with F1 scores surpassing 90% in certain instances, and yielded promising outcomes in metrics such as IoU and Frechet Inception Distance. References [84,85] researched denoising PET images utilizing denoising diffusion probabilistic models. Their studies demonstrated impressive denoising capabilities, as evidenced by high values of SSIM and PSNR. These metrics indicate that the models effectively remove noise while keeping important image information. Reference [87] employed a Variable-Order Fractional Diffusion Model on imaging modalities such as MRI, CT, and X-ray. Their study showed consistent outcomes with high PSNR and low MSE, affirming the efficacy of diffusion models in noise reduction across diverse imaging modalities. Reference [88] utilized an unsupervised diffusion probabilistic model to process retinal OCT images in their study. The results demonstrated significant improvement in denoising, as seen by the high SNR and PSNR values.

Regarding RQ1, which focuses on the accuracy of diffusion models in health informatics denoising tasks, the models exhibit robust performance, particularly in improving image quality across different modalities such as MRI, CT, PET, and X-ray. The feature map denoising diffusion and denoising diffusion probabilistic models have demonstrated significant efficacy, attaining high PSNR and SSIM values. These values are crucial for preserving the structural integrity of medical images. Regarding RQ2, the research employed a wide variety of datasets, encompassing publicly available and privately sourced data and several imaging methods. Nevertheless, although these datasets exhibit diversity, their thoroughness could be enhanced by incorporating a more comprehensive range of diseases and imaging situations. Regarding RQ3, the issue of explainability needs to be given more attention in these denoising models. Only a small number of studies have included methods to interpret the denoising process. The absence of emphasis on explainability may impede the therapeutic acceptance of these theories. RQ4 emphasizes the participation of clinicians, which is observed in around 33% of the studies, namely, those that utilize PET and X-ray imaging. This indicates that although there is some level of cooperation between researchers and healthcare professionals, it is not yet prevalent. Ultimately, RQ5 focuses on the difficulty of implementing real-time deployment, and it is evident that this objective has not been achieved. None of the studies have successfully implemented these models in real time, highlighting the necessity for further study to enhance their efficiency for quicker processing and incorporation into clinical procedures.

### 5.4. Generation

Generation processes in health informatics are essential for producing synthetic data of superior quality, improving existing medical imaging, and completing incomplete datasets by filling in missing information [21]. Diffusion models are highly useful in these tasks because they can provide realistic and highly detailed images in different modalities, including MRI, CT, and X-ray [89,90,91]. This feature is crucial for super-resolution imaging, inpainting, and data augmentation applications. These applications can significantly enhance diagnostic accuracy and facilitate the creation of more resilient machine-learning models by expanding the variety and quantity of training data. Diffusion models have a crucial impact on clinical practice and medical research progress by creating synthetic images that closely mimic actual medical data.

Table 8 shows that the examined papers demonstrate the adaptability of diffusion models in generating tasks across various medical imaging modalities. Models such as Conditional Diffusion Probabilistic and cross-guided diffusion have been used in tasks like MRI generation and chest X-ray inpainting. These models have achieved remarkable results, including high PSNR and SSIM scores, as demonstrated in [89,92]. The effectiveness of other models, such as the class-conditional diffusion and Infinite Diffusion–Hierarchical Diffusion, in generating high-quality images from retinal OCTs, histopathology slides, and ultrasound data has been demonstrated by [21,93]. Metrics such as FID and IS reflect the high fidelity of the generated images. Key metrics indicate solid performance, with FID values spanning 0.48–206.25 and recent approaches [89,92] achieving scores below 1 for image generation. The cross-guided diffusion model attains the best PSNR values of 40.16 (Chest X-ray14), 39.51 (SILVER07), and 42.48 (ACDC), and in most works, the overall SSIM scores are higher. The research highlights the significance of model flexibility, precisely the effectiveness of Deformable Diffusion and Conditioned Latent Diffusion in producing images that improve diagnostic capacities for various medical disorders and imaging modalities.

Regarding RQ1, the efficacy of diffusion models in generating tasks in health informatics is apparent. Models such as conditional denoising diffusion probabilistic and cross-guided diffusion have consistently produced high-quality outcomes in diverse imaging modalities like MRI, CT, and X-ray. The consistently high PSNR, SSIM, and FID scores seen in these investigations demonstrate the models’ capacity to produce realistic and medically valuable images. RQ2 focuses on the variety of datasets used in these studied, including a wide range of publicly available and privately owned datasets. These datasets covered several types of imaging, such as retinal OCTs and histopathology slides, ensuring that the models were tested in diverse settings. Nevertheless, the inclusiveness of these datasets could be further improved by incorporating a wider range of disease disorders. Regarding RQ3, the issue of explainability is still a notable deficiency since none of the research explicitly provides tools to clarify the process of making and generating judgments. This exclusion can potentially restrict the adoption of the models in clinical settings. RQ4 examines the level of clinician participation, which is significant in this field as almost 66% of the studies indicate active cooperation with healthcare providers. This participation is essential to guaranteeing that the produced photographs adhere to therapeutic standards and are pertinent to healthcare applications. Furthermore, RQ5 emphasizes that integrating these generation models into live clinical settings remains a substantial barrier as none of the examined research has successfully achieved real-time deployment. This highlights the necessity for ongoing research into enhancing these models for immediate execution and smooth incorporation into healthcare systems.

### 5.5. Reconstruction

Reconstruction in health informatics is essential for enhancing the quality of medical images, especially when data is inadequate or damaged, such as in rapid MRI or low-dose CT scans [97]. Diffusion models have demonstrated significant potential in this field by recreating high-fidelity images from data that is either under-sampled or affected by noise [97,98]. As a result, they improve the diagnostic effectiveness of medical imaging. This feature is essential for facilitating expedited, secure, and precise medical diagnosis, particularly in cases where time is of the essence or resources are limited. Diffusion models are important tools in modern healthcare as they aid in preserving crucial anatomical characteristics during reconstruction activities.

Table 9 illustrates that reviewed papers emphasize the efficacy of several diffusion models in reconstructing high-fidelity images from various datasets, such as brain MRI, knee MRI, and CT images. The models AdaDiff and MC-DDPM, employed by [97,99], respectively, have shown outstanding performance, especially in terms of PSNR and SSIM, on datasets such as IXI and fastMRI. These models proficiently manage under-sampled MRI data, guaranteeing the preservation of crucial diagnostic information. References [98,100] demonstrated impressive outcomes using the SSDiffRecon and SMRD models. These models were particularly effective at reconstructing brain and knee MRI images with minimum loss of detail, especially when dealing with higher acceleration rates. References [24,101] expanded diffusion models to CT images and multi-coil MRI data, showcasing the adaptability of these models across various imaging techniques and complexities. Reference [102] also introduced HFS-SDE, a promising method for knee MRI reconstruction, which exhibited strong NMSE and SSIM values. A concise qualitative analysis indicates that brain MRI reconstruction methods consistently demonstrate high-fidelity performance. Reference [97] reports PSNR values in the range of 41.9–42.6 and SSIM values of 98.9%, whereas another study [98] achieves PSNR values of up to 45.9 and SSIM 99.0%, indicative of near-perfect structural preservation on clean datasets. On the more challenging fastMRI benchmark, reported PSNR values typically fall within 36–40.

When examining RQ1, the evaluated diffusion models for reconstruction tasks in health informatics demonstrate significant efficacy, especially in dealing with under-sampled and noisy data. This is supported by robust PSNR and SSIM scores observed in different MRI and CT imaging modalities. This highlights their capacity to enhance diagnostic precision in practical clinical environments. RQ2 demonstrates that these models have been tested on several datasets, such as fastMRI and IXI, encompassing brain, knee, and abdominal imaging. However, the inclusiveness of these datasets might be enhanced by incorporating a broader spectrum of disorders. Regarding RQ3, the studies do not prioritize explainability as only a few models have elements that would clarify the reconstruction process. This lack of transparency could hinder their acceptance in clinical settings. RQ4 demonstrates some clinician engagement, namely, in research centered around MRI reconstruction. However, this involvement is constrained, indicating the necessity for further cooperation between creators of models and healthcare practitioners. RQ5 emphasizes the ongoing difficulty in deploying models in real time as none of the studies have successfully implemented real-time functionality. This emphasizes the necessity for additional research to enhance these models for quicker processing and seamless integration into clinical processes.

### 5.6. Segmentation

Segmentation allows for accurate identification of anatomical structures and diseased areas in medical images [105]. This is vital for diagnosis, treatment planning, and disease monitoring. Diffusion models have become potent instruments for segmentation because they can capture intricate details and structures in many imaging modalities, including MRI, CT, and ultrasound [22,105,106]. Their use in segmenting tissues, cancers, and lesions enables more precise and dependable analysis, substantially enhancing therapeutic outcomes and facilitating personalized medicine approaches.

Table 10 shows that References [107] and [22,108] consistently show the strong capabilities of diffusion models in various medical imaging segmentation tasks. Reference [107] employed models such as TGEDiff and DBEF-Net to analyze endoscopic and dermoscopic datasets like Kvasir-SEG and ISIC2016. The authors achieved impressive F1 scores and Dice coefficients.

The performance of this study is similar to the findings of [109,110] who used DIM-UNET and DiffABAL on datasets such as ISIC2018 and BCSS. The segmentation accuracy remained consistently high across various imaging modalities. In addition, References [106,111] utilized diffusion models such as CIMD and TransDiff on intricate datasets, such as lung CT and brain MRI, demonstrating robust metrics such as Dice scores and specificity, particularly in datasets like LIDC-IDRI and BraTS2021. In addition, References [112,113] emphasized the efficacy of diffusion models such as CorrDiff+ and MedSegDiff in managing brain MRI and fundus images, attaining exceptional segmentation outcomes on datasets like BraTS2020 and Refuge-2. The overall qualitative analysis reports that diffusion-based and segmentation-based frameworks showcase predominant performance on public benchmarks. For example, exceptional Dice scores (0.998 in pathological data [109], 0.939–0.906 in CRAG/GlaS/RINGS [107], 0.92 in kidney CT [111], and 0.909–0.90 in BraTS variants [110,112,113] reflect superior segmentation performance. However, performance drops slightly lower for ultrasound (77.95 F1 on BUSI [22], 86.1 Dice on DDTI [113]) and complex lesions (0.61 Dice for certain pelvic structures). The consistent and reliable performance observed in these studies highlights the versatility of diffusion models in addressing various segmentation challenges, ranging from intricate brain structures to complicated pathological regions. This confirms their potential to outperform traditional methods in clinical applications.

The efficacy of diffusion models in segmentation tasks is demonstrated by the notable Dice coefficients, F1 scores, and specificity metrics achieved across diverse imaging modalities, such as brain MRI, dermoscopic images, and endoscopic images, regarding RQ1. These models have proven their capacity to precisely define intricate anatomical structures and diseased areas, which is essential for clinical purposes. In relation to RQ2, the research employed a diverse group of datasets, primarily public datasets such as ISIC, BraTS, and Kvasir, to ensure a wide range of testing scenarios. However, the comprehensiveness of the studies might be improved by incorporating a greater variety of diseases and patient demographics. The findings of RQ3 indicate that the research examined should have prioritized the issue of explainability as only a small number of studies included explanation of the segmentation process. Moreover, those studies highly rely on visualization-based explainability methods, including model attention maps and uncertainty variance maps. This lack of interpretability features may hinder the widespread use of these methods in clinical settings. RQ4 demonstrates that approximately 50% of the studies include the participation of clinicians, especially those that concentrate on brain MRI and lung CT. This suggests some level of collaboration between researchers and healthcare professionals. However, there is still potential for greater involvement to ensure model development is more closely aligned with clinical requirements. Finally, RQ5 emphasizes the persistent difficulty of deploying models in real time as only two studies [105,113] have reported actual real-time implementation. This emphasizes additional research’s need to enhance these models for quicker processing and smooth integration into clinical processes.

**Table 10:** Diffusion models in segmentation. ✓ denotes presence in the study, and × denotes absence in the study.

Reference	Dataset Name	Dataset Availability	Dataset Type	Model	Results	XAI	Clinician Involvement	Real-Time Implementation
[22]	(a) ISIC2018	Public	Dermoscopic	DIM-UNet	F1: 91.41; IoU: 84.51	×	×	×
	(b) BUSI		Ultrasound		F1: 77.95; IoU: 64.27			
	(c) Kvasir dataset		Endoscopic		F1: 87.39; IoU: 78.21			
[105]	Local dataset	Private	Pelvic radiograph	DDPM	(OF; GT; LT) DSC: (0.90; 0.84; 0.61) FID: 7.2; IS: 210	×	✓	✓
[112]	BraTS2020	Public	Brain MRI	Diffusion-based	Dice: 0.909 HD95: 5.178 Jaccard: 0.845	✓	✓	×
[106]	(a) Lung lesion segmentation (LIDC-IDRI)	Public	Lung CT	CIMD	(GED; CI; MaxDice) (0.321; 0.759; 0.915)	×	✓	×
	(b) Bone surface segmentation (B-US)		Ultrasound		(0.295; 0.7578; 0.889)			
	(c) Multiple sclerosis lesion segmentation (MS-MRI)		MS-MRI		(0.733; 0.560; 0.562)			
[107]	(a) GlaS	Public	Pathological	Diffusion-based	(Test A; Test B) Dice: (0.939 ± 0.060; 0.889 ± 0.069) F1: (0.941 ± 0.039; 0.893 ± 0.073)	×	×	×
	(b) CRAG				F1: 0.853 ± 0.054 Dice: 0.906 ± 0.043			
	(c) RINGS				Precision: 0.893 ± 0.096 Dice: 0.904 ± 0.091			
[114]	(a) Kvasir-SEG	Public	Endoscopic	TGEDiff	(F1 score; mIoU; Precision; Recall) (90.70; 84.89; 91.66; 92.53)	✓	×	×
	(b) Kvasir-Sessile				(84.3; 77.05; 92.26; 83.81)			
	(c) GLaS				(88.51; 80.69; 90.47; 88.84)			
[108]	(a) ISIC2016	Public	Dermoscopic	DBEF-Net	(F1; IoU; Precision; Recall) (93.79; 88.31; 95.89; 91.79)	×	✓	×
	(b) ISIC2017				(86.87; 76.79; 94.8; 80.17)			
	(c) PH2				(93.1; 87.09; 89.84; 96.61)			
	(d) GLAS		Microscopic		(87.03; 77.05; 88.78; 85.36)			
[111]	(a) BraTS2021	Public	Brain MRI	TransDiff	(Dice; SEN; IoU; HD95) (90.1; 91.3; 82.0; 23.6)	×	×	×
	(b) ISIC2017		Dermoscopic		(89.6; 88.3; 81.2; 21.7)			
	(c) KiTS19		Kidney CT		(92.1; 92.5; 85.4; 22.5)			
[109]	(a) BCSS	Public	Pathological	DifABAL	Dice: 99.8% (with 100% labeled data)	×	✓	×
	(b) CXR		X-ray					
	(c) ISIC2018		Dermoscopic					
[113]	(a) Refuge-2	Public	Fundus	MedSegDiff-L	(Dice; IoU) (86.9; 78.5)	✓	✓	✓
	(b) BraTS2021		Brain MRI		(89.9; 82.3)			
	(c) DDTI		Ultrasound		(86.1; 79.6)			
[110]	(a) BraTS2020	Public	Brain MRI	CorrDiff+	(ET; WT; TC) Sensitivity: (83.82; 92.73; 85.14) Specificity: (99.98; 99.93; 99.97) mDSC: (86.78) mHD95: (8.75)	×	×	×
	(b) BraTS2019				mDSC: (87.09) mHD95: (4.46)			
	(c) Jun Cheng				DSC: (92.81) Sensitivity: (92.13) Specificity: (99.94) HD95: (3.04)			

### 5.7. Super-Resolution

Super-resolution is a technique that improves the quality of low-resolution medical images by converting them into high-resolution equivalents [108]. This is crucial for achieving precise diagnosis, planning treatments, and conducting research. Diffusion models have demonstrated considerable promise by efficiently restoring fine details from low-quality inputs, specifically in brain MRI, knee MRI, and micro-CT images [115,116]. This feature is crucial for increasing the clarity of medical images, enabling healthcare practitioners to identify and examine more intricate elements that may go unnoticed in lower-resolution images. As a result, it improves the accuracy of diagnoses and ultimately leads to better patient outcomes.

Table 11 depicts the studies [117,118,119] demonstrating the efficacy of diffusion models in super-resolution tasks across various datasets, including brain MRI datasets like IXI and fastMRI. Reference [117] applied ASSRDM, achieving high PSNR and SSIM scores on these datasets comparable to the results of [119], with their InverseSR model also yielding strong SSIM and PSNR metrics. The analysis of the results shows that, on brain MRI benchmarks, the method delivers outstanding performance with very high PSNR (37.53–37.66) and nearly perfect SSIM (0.986–0.988). It also demonstrates strong clinical applicability, achieving PSNR values of 33.85–35.47 and SSIM values up to 0.9607 across tumor and pelvic MRI datasets. Reference [118] applied a DDPM-based model to the Amsterdam Open MRI Collection, achieving a PSNR of 24.63 and an SSIM of 0.7847, underscoring the model’s effectiveness in enhancing brain MRI resolution. Additionally, References [116,120] extended the application of diffusion models to pediatric brain MRI and the Harvard Medical School Database, with [116]’s Cas-DiffCom and [120]’s TFS-Dif models showing substantial improvements in image quality, as reflected in their respective metrics. References [121,122] further broadened the scope by applying diffusion models like ResDiff and DiffMSR to general and clinical datasets, including FFHQ and clinical pelvic MRI, achieving notable PSNR and SSIM values that underscore the models’ robustness across different anatomical sites. References [115,123] focused on more specialized applications, such as micro-CT and knee MRI, with both studies reporting significant correlations and high fidelity in the reconstructed images, thereby confirming the versatility and reliability of diffusion models in diverse super-resolution tasks.

In relation to RQ1, diffusion models have demonstrated significant efficacy in super-resolution tasks in the field of health informatics. These models have achieved high PSNR and SSIM scores in various medical imaging modalities, including brain MRI, knee MRI, and micro-CT. This indicates their robust ability to improve image resolution and enhance fine details. RQ2 demonstrates that these models have undergone testing on a wide array of datasets, such as public datasets like IXI, fastMRI, and clinical brain MRI. However, the inclusiveness of the testing might be enhanced by integrating a greater variety of patient demographics and pathologies. Regarding RQ3, the topic of explainability has not been thoroughly investigated. Most studies have yet to include mechanisms to clarify the super-resolution process, which could hamper the clinical use of the models. RQ4 demonstrates partial clinician engagement, notably in brain MRI and knee MRI studies, suggesting a modest degree of collaboration between researchers and healthcare practitioners. However, there is a need for increased involvement to guarantee that the models are in line with clinical requirements. RQ5 emphasizes the difficulty of real-time deployment, with only one study successfully implementing it.

### 5.8. Translation

Translation roles in health informatics serve a crucial role in producing excellent images by converting them from one modality to another [125]. This includes transforming CT scans into MRI images or low-dose images into their full-dose equivalents. Diffusion models have demonstrated particular importance in this field by allowing the creation of medically valuable images that maintain crucial diagnostic information [126]. This feature is essential in instances when obtaining specific imaging techniques is difficult or where reducing the dosage is important to ensure patient safety.

The experiments conducted in [127,128] and Reference [125] provide evidence of the effectiveness of diffusion models in different translation tasks, including converting CT to MRI and enhancing images in lumbar spine and chest radiography depicted in Table 12. Reference [127] applied the Frequency Domain Decomposition Method (FDDM) on the SynthRAD2023 dataset and obtained impressive SSIM and PSNR scores. This performance is similar to the results achieved in [128] using ContourDiff on various spine and hip datasets. Both studies demonstrated excellent accuracy in preserving the structure. The overall qualitative analysis reports that diffusion models improve cross-modality image synthesis such as MRI-to-CT, T1-to-T2/FLAIR, X-ray soft-tissue or bone suppression, and CT-to-CBCT on primarily public datasets. The strongest performance is observed in intra-modality or similar-contrast scenarios: SynDiff [23] attains high PSNR (30–35.47) and SSIM (94–96.98%) for brain MRI contrast translation, while CMDM [125] yields very high PSNR (44) and almost ideal SSIM (0.99) for chest X-ray decomposition and sparse-view low-dose CT synthesis. In contrast, the cross-modality results are more heterogeneous and generally inferior (PSNR 24–28, SSIM 0.805–0.908, FID up to 94.8 in FGDM [129]; FID 71–143 in TGDM [130]), underscoring the difficulty of maintaining anatomical information.

Reference [125] expanded this technique to dual-energy chest X-rays, employing CMDM to attain outstanding image quality characterized by high PSNR and SSIM values. In addition, References [131,132] utilized models such as MS-SPADE and MIDiffusion on brain and pelvic imaging datasets, demonstrating consistent and excellent performance across various modalities and datasets, highlighting the models’ resilience and flexibility. The research conducted in [23,130] has provided more evidence of the capabilities of diffusion models in converting information between MRI and ultrasound, as well as MRI and CT. These studies have shown significant enhancements in fidelity and resolution. These findings collectively demonstrate the ability of diffusion models to produce clinically feasible translations that have the potential to substitute or complement conventional imaging techniques.

Regarding RQ1, diffusion models have demonstrated significant efficacy in translation tasks in health informatics. They have achieved excellent SSIM and PSNR scores in functions such as CT to MRI conversion and dose reduction, suggesting their robust capacity to improve image quality across different modalities. RQ2 demonstrates that these models have undergone extensive testing on a diverse set of datasets, encompassing both public and private datasets such as SynthRAD2023, BraTS2021, and AAPM low-dose CT. However, it would be beneficial to include a wider range of imaging circumstances and patient demographics to enhance the variety. In relation to RQ3, the emphasis on explainability is not given much importance as none of the studies include aspects that allow for interpretation. RQ4 demonstrates a modest level of clinician participation, especially in studies that concentrate on spine and brain imaging. This suggests a certain degree of collaboration between researchers and healthcare practitioners. However, there is a need for improvement in order to guarantee clinical significance. RQ5 emphasizes that the implementation of real-time systems is still a significant obstacle as only two studies incorporated their study into real-time systems.

### 5.9. Overview of the Domains

The eight explored domains in medical imaging exhibit a combination of strengths and weaknesses when evaluated in terms of their applicability regarding explainable artificial intelligence, clinician involvement, and real-time implementation. Despite the noteworthy advancements in the application of diffusion-based models, considerable gaps remain in achieving fully functional, real-time, and clinically integrated solutions across these domains.

For instance, diffusion models demonstrate significant promise in anomaly detection, particularly due to their ability to identify irregularities in complex imaging modalities such as MRI. Nonetheless, anomaly detection faces challenges primarily related to the limited integration of XAI despite its higher usage in this domain (26.67%) compared to others. The lack of diverse datasets further constrains generalizability as most studies predominantly rely on MRI data. Moreover, real-time implementation is absent mainly in this domain across most studies. In classification, diffusion models exhibit strong predictive power across various imaging modalities, including X-rays, retinal optical coherence tomography (OCT), and histopathology images. However, this domain needs more clinician involvement than others, and XAI features are also limited, raising concerns regarding the interpretability and trustworthiness of model outputs among healthcare professionals. Additionally, no real-time applications have been reported in this classification.

Conversely, the denoising domain has demonstrated notable success with diffusion models, particularly in enhancing image quality in MRI, CT, and PET scans. Although these models perform effectively in denoising tasks, their clinical applicability is somewhat impeded by the limited use of XAI. Approximately 33% of studies in this domain included clinician involvement, which is relatively low given the critical role of denoising in improving diagnostic accuracy. Furthermore, denoising models face challenges in real-time deployment. Similarly, the generation domain also lacks XAI features, with none of the reviewed studies incorporating explainability tools, which presents a significant barrier to clinical adoption. State-of-the-art generation tasks exist in other models, and in our future study section, we detail how future researchers can improve, including XAI [133,134]. Although clinician involvement in the generation domain is relatively high (17.95%), real-time implementation remains a major challenge as studies still need to integrate these models into live clinical workflows successfully.

In the reconstruction domain, diffusion models are primarily utilized to recreate high-fidelity images from under-sampled or noisy data, particularly in MRI and CT imaging. Reconstruction tasks have seen better integration of XAI (26.67%). However, similar to the segmentation domain, while clinician involvement is evident, it remains inconsistent in practical settings. This domain also faces substantial challenges with real-time implementation. Super-resolution techniques have benefited from the application of diffusion models; however, despite their performance, super-resolution models suffer from a lack of XAI, posing concerns regarding their adoption in clinical contexts. Real-time deployment continues to be challenging as none of the reviewed studies in this domain have incorporated real-time functionality.

Lastly, translation tasks involving converting images from one modality to another (e.g., CT to MRI) have shown significant potential with diffusion models. However, similar to other domains, translation tasks lack XAI features, and it is shown that XAI improves the translation tasks. While clinician involvement is relatively high (17.95%), the absence of real-time capabilities limits the clinical impact of these models as translation tasks often necessitate immediate application in medical workflows. Section 6 broadly discusses the overview in terms of the RQs.

## 6. Discussion of Research Questions

The effectiveness of diffusion models in health informatics exhibits substantial variation across eight key domains. In the literature analysis in Section 5, we have comprehensively discussed these eight domains. In this section, we present the linkage between the proposed research questions and address their answers to justify our findings sequentially. The first RQ1 synthesizes diffusion model performance across health informatics tasks; RQ2 examines dataset diversity in broader health data; RQ3 evaluates explainability techniques and their effects on interpretability and transparency; RQ4 analyzes the roles, clinical relevance and implementation of clinicians in the real-world; and RQ5 addresses the key challenges and future directions for integrating diffusion models into health informatics. In our findings for RQ1, we determined the conditional-guided diffusion and multi-scale diffusion models to demonstrate exceptional performance in anomaly detection and segmentation tasks, especially when applied to MRI datasets, resulting in remarkable levels of specificity and accuracy. Conditioned, multi-scale diffusion models guide denoising while preserving key anatomical structures and boundaries, suppressing irrelevant noise and highlighting features crucial for delineating anomalies or regions of interest. Consequently, these models perform strongly in both segmentation and anomaly detection, which also aligns with observations obtained from RQ1. Latent Diffusion models excel in classification and reconstruction tasks, effectively using various datasets such as X-rays and MRI scans to improve image quality and enhance diagnostic accuracy.

Denoising diffusion probabilistic models are highly efficient in removing noise from images, but Super-Resolution Diffusion Models primarily improve picture resolution, particularly with MRI data. Nevertheless, score-based generative models and cross-modal diffusion models exhibit promise in generation and translation tasks, yet they require more varied datasets to enhance their generalization ability.

**Figure 8:**
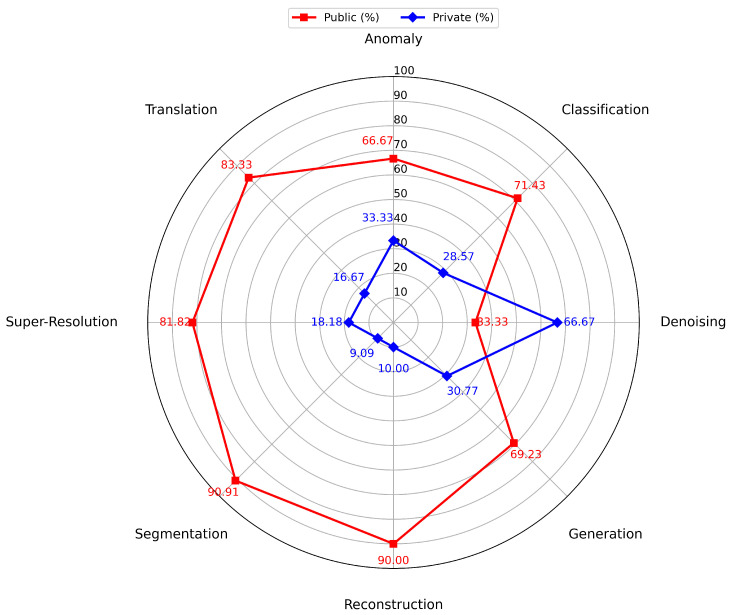
Analysis of RQ2. Radar chart illustrating the comparative analysis of public and private dataset usage across various medical imaging tasks in diffusion model research.

Our RQ2 assesses the diversity of the dataset, although it is currently limited, especially in domains such as anomaly detection and generation, which mainly depend on MRI and synthetic data. Latent Diffusion models rely on different datasets to perform classification and reconstruction tasks. However, their heavy dependence on publicly accessible datasets (Figure 8) that are specialized to a certain modality, such as MRI, indicates a requirement for more extensive and diverse sources of data. Incorporating a wider range of data types, such as PET and ultrasound, into the dataset could significantly improve the reliability and usefulness of diffusion models in all fields. The reason behind the performance in private and public datasets is that public medical datasets are typically larger, better curated, and more standardized, offering richer, more diverse training data. In contrast, private datasets are smaller and often lack diversity. Since diffusion models need extensive data to learn forward and reverse noise processes, limitations like small sample size hinder training and generalization. This analysis highlights the significance of optimizing both the models and the datasets they are trained on to exploit their potential in health informatics fully.

**Figure 9:**
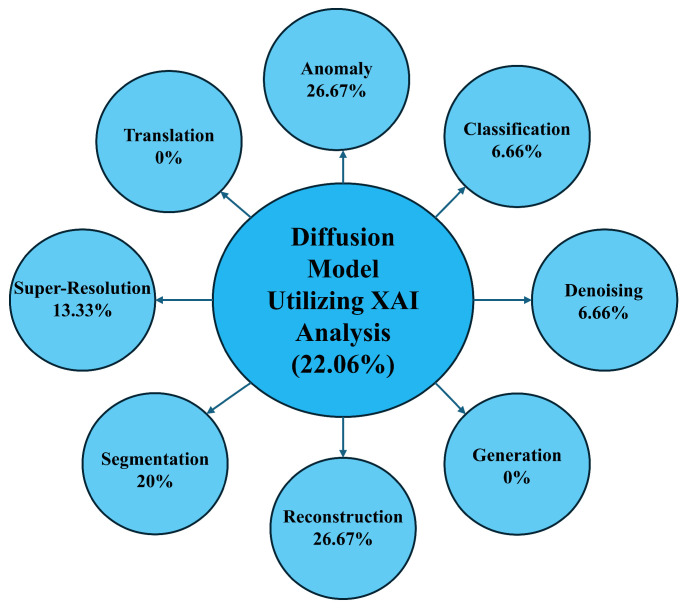
Analysis of RQ3. Proportional distribution of diffusion model studies that incorporate explainable AI (XAI) techniques across different imaging tasks.

The analysis of RQ3 shows that the integration of explainability (XAI) into diffusion models is present in 22.06% of the reviewed papers (Figure 9). However, the distribution across various tasks reveals gaps—XAI is more frequently applied in anomaly detection (26.67%) and reconstruction (26.67%) but is absent in generation and translation tasks. This uneven distribution highlights the need for more consistent incorporation of XAI features, especially in tasks where transparency is critical for clinical decision-making. Figure 8 depicts the whole scenario. According to the studies reviewed, explainability was primarily implemented using attention maps and pixel-level feature analyses to interpret model prediction. Moreover, some studies used different statistical and mathematical analysis to clarify the model predictions. Moreover, it is observed that XAI methods are mostly used in tasks where interpretability directly affects clinical decisions such as lesion segmentation or anomaly localization because clinicians must see which regions or features drive model outputs to ensure trust and patient safety. In contrast, generation and denoising tasks are validated mostly on visual information rather than clinically grounded reasoning, reducing the incentive to add explainability.

**Figure 10:**
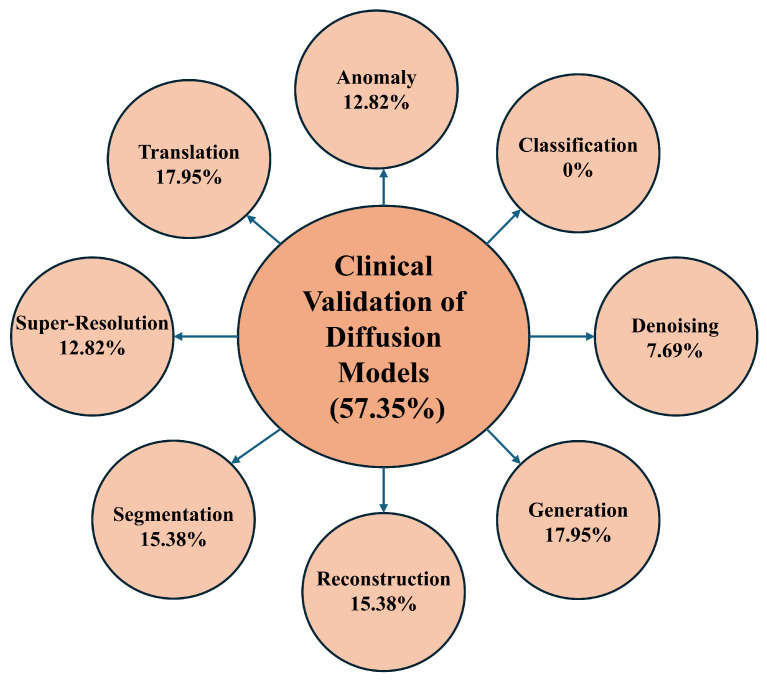
Analysis of RQ4. Distribution of diffusion model studies that include clinical validation across various imaging tasks.

Our RQ4 concerns the clinician involvement reported in 57.35% of the studies (Figure 10), with the highest presence in generation and translation tasks (17.95% each), illustrated in Figure 9. The higher clinician involvement also underscores direct clinical impact of these tasks on diagnosis, requiring expert validation to ensure anatomical plausibility and patient safety. Despite this, there are areas like classification where clinician involvement could be more present. A key reason for the limited involvement with classification tasks is that their outputs are mostly evaluated using performance metrics. However, this uneven involvement suggests that while considerable effort exists to include clinicians in the research process, further collaboration is necessary to ensure that the models are clinically relevant and aligned with real-world healthcare needs.

**Figure 11:**
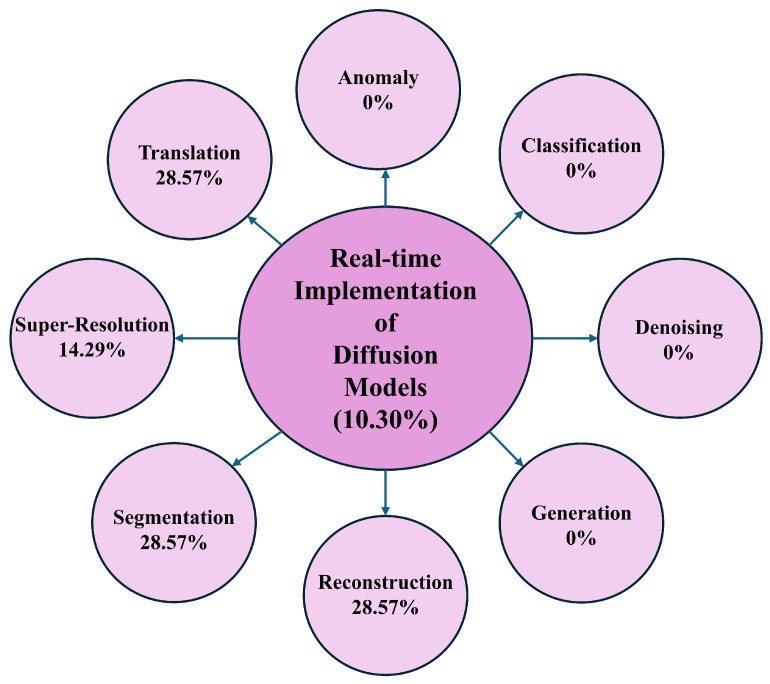
Analysis of RQ5. Distribution of real-time implementation of diffusion models across medical imaging tasks.

The analysis of RQ5 in this review shows that the real-time implementation of diffusion models in health informatics remains a significant challenge, with only 10.30% of the papers addressing it (Figure 11). Real-time applications are mainly found in reconstruction, segmentation, and translation tasks (28.57% each) but are completely lacking in anomaly detection, classification, and generation tasks. This indicates that achieving real-time performance is still a significant hurdle, requiring further advancements in computational efficiency and optimization for clinical environments.

Real-time deployment of diffusion models for image synthesis or denoising is difficult because iterative sampling can require thousands of steps, causing latency that breaks strict real-time constraints. These continuous forward and reverse diffusion steps accumulate more memory and require more computational power for execution. Moreover, studies utilizing 3D medical images or video samples demand higher processing resources, therefore being significantly challenging for real-time deployment. As a result, tasks like segmentation or classification, conditioning, and task-specific optimizations can reduce steps and approach real time, but fully unconstrained generation without conditional guidance remains much slower at inference. Consequently, for generation, denoising, reconstruction, and anomaly detection, real-time usage is slower, which also aligns with our RQ5 findings.

## 7. Future Research Directions

After conducting an extensive analysis, we suggest 12 Future Research Questions (FRQs) that must be investigated by future scholars in the field of diffusion models for health informatics. The areas of attention for further inquiry include RQ3–5, which pertain to the integration of explainability, clinician engagement, and real-time implementation.

The primary objective of these forthcoming research inquiries is to ascertain and surmount pivotal obstacles in the domain, focusing on augmenting efficiency, diversifying datasets, elucidating model interpretability, and bolstering clinical significance. The following FRQs outline research avenues that can be taken to optimize diffusion models to develop more robust and therapeutically applicable solutions. Table 13 provides a systematic summary of the main topics that need to be further explored to enhance the effectiveness of diffusion models in health informatics. The FRQs are derived from a comprehensive examination of 68 publications and emphasize the urgent requirements within the area. The purpose of these questions is to tackle significant problems, such as reducing the inference time and memory usage while maintaining accuracy and efficiency, especially in real-time clinical applications. Additionally, they aim to enhance image quality in datasets with limited information, such as CT, MRI, and PET scans, by incorporating advanced reconstruction algorithms and augmentation techniques to minimize artifacts and enhance image fidelity. Furthermore, the FRQs seek to address various imaging conditions with minimal (model) re-training by advocating for modular model architectures and adaptive algorithms capable of handling diverse scenarios, including non-rigid motion corrections.

The emphasis on semi-supervised learning techniques highlights the increasing significance of including labeled and unlabeled data to enhance the generalization of models. Furthermore, it is crucial to incorporate clinicians in order to maintain the relevance and applicability of diffusion models to real-world clinical circumstances. As per the FRQs, future studies should focus on leveraging auxiliary tasks and multi-scale feature aggregation to enhance segmentation model performance and versatility. Additionally, optimizing feature grouping strategies will be key to managing varying feature correlations and importance across different channels. The study routes also propose the utilization of modular model architectures, adaptive techniques for motion correction, and innovative feature grouping strategies to improve model performance across different workloads. The text discusses the difficulties of balancing computational economy and image quality, particularly in the context of 3D super-resolution. Thus, addressing the trade-off between image quality and computational efficiency in 3D super-resolution models requires innovations in both super-resolution techniques and computational optimization methods. Finally, developing end-to-end solutions that integrate data preprocessing within the model architecture and building on one-to-many modality translations will further advance diagnostic accuracy and address practical clinical challenges. Future research on medical diffusion modeling is likely to converge on unified, large-scale, clinically grounded frameworks. Our FRQs also highlight the integration of diffusion models with foundation architectures to improve cross-modal generalization and parameter-efficient adaptation. Moreover, physics-informed models, federated learning, and state-space backbones can enable scalability aligned with clinical and operational constraints. Together, these advanced methods will produce more robust, data-efficient diffusion models applicable across diverse clinical domains. Overall, our FRQs aim to address the existing deficiencies in diffusion model research, hence creating opportunities for advancements and providing future research paths that can significantly enhance the healthcare industry.

Finally, this study highlights the transformative potential of the diffusion models in health informatics, along with their innovative contribution to the advanced engineering domain. Furthermore, this work significantly upholds the advancing AI-based engineering applications in the medical field, ultimately outlining their impact and opportunities to improve patient outcomes and optimize overall healthcare treatment.

## 8. Conclusions

This review emphasizes the substantial influence of diffusion models in many medical imaging applications, offering a comprehensive examination of their functions in anomaly detection, classification, denoising, generation, reconstruction, segmentation, super-resolution, and translation. Through an examination of the level of clinician participation, the application of XAI, and the integration of real-time implementation, we have found areas of proficiency and potential for improvement in existing research. Although diffusion models show promise, our research highlights the necessity for greater incorporation of XAI (found in just 22.06% of publications) and higher emphasis on real-time implementation (seen in just 10.30% of studies). Furthermore, the significant participation of clinicians in more than half of the papers (57.35%) highlights the crucial significance of collaborative endeavors between AI researchers and healthcare practitioners.

In order to promote future progress, we suggest twelve future research inquiries to improve XAI, the integration of clinicians, and the real-time capabilities of diffusion models. This review provides a significant resource for scholars and practitioners in health informatics, presenting the current status of diffusion models and suggesting strategic pathways for further advancement. Overall, this study clearly demonstrates the impact of the AI-based advanced engineering framework in the medical field, and the findings with future direction will provide adequate guidelines to optimize its utilization and provide better outcomes.

## Figures and Tables

**Table 1:** The benchmark comparison with state-of-the-art review papers. ✓ denotes presence in the study, and × denotes absence in the study.

Paper	Performance Analysis	Dataset Analysis	Taxonomy	XAI Analysis	Clinician Involvement Analysis	Future Direction
[26]	✓	✓	✓	×	×	✓
[29]	✓	✓	✓	×	✓	✓
[27]	×	×	✓	×	×	✓
[30]	×	×	✓	×	×	✓
[31]	×	×	✓	×	×	×
[32]	✓	✓	✓	×	×	×
[15]	×	✓	✓	×	×	×
[33]	×	×	×	×	×	×
[28]	×	✓	✓	×	×	✓
[34]	✓	×	×	×	×	×
[35]	×	×	✓	×	×	✓
[36]	×	×	✓	×	×	✓
[37]	×	✓	✓	×	×	✓
Our Review	✓	✓	✓	✓	✓	✓

**Table 2:** Comparison of the different types of diffusion models.

Technique	Probabilistic Models	Score-Based Models	Stochastic Differential Equations (SDEs)
Characteristics	Emphasize the modeling uncertainty.	Use score functions to estimate data distributions.	Incorporate inherent randomness directly into model systems.
Working Technique	Employ probabilistic distributions to represent data, especially using Markovian or Bayesian methods.	Learn the gradient (score) of the data distribution, often using neural networks.	Model the evolution of systems over time with noise.
Forward Process	$\begin{array}{c} q(x_{t}|x_{t-1})=\\ N(x_{t};\sqrt{1-\beta_{t}}\cdot x_{t-1},\\ \beta_{t}\cdot I),\\ \forall t\in \{1,\ldots ,T\} \end{array}$	$\begin{array}{c} x_{t}=x_{t-1}+\\ \sqrt{\sigma_{t}^{2}-\sigma_{t-1}^{2}}\cdot z_{t},\\ z_{t}∼N\left(0,I\right) \end{array}$	dx=f(x,t)dt+σ(t)dω
Reverse Process	$\begin{array}{c} p_{\theta }\left(x_{t-1}|x_{t}\right)=\\ N(x_{t-1};\mu_{\theta }\left(x_{t},t\right),\\ \Sigma_{\theta }\left(x_{t},t\right)) \end{array}$	$x_{t-1}=x_{t}+\left(\sigma_{t}^{2}-\sigma_{t-1}^{2}\right)\nabla_{x_{t}}\log q\left(x_{t}\right)+\left(\sqrt{\sigma_{t}^{2}-\sigma_{t-1}^{2}}\right)z_{t}$	$\begin{array}{c} dx=[f\left(x,t\right)-\sigma {\left(t\right)}^{2}\\ \nabla_{x}\log p_{t}\left(x\right)]dt+\\ \sigma \left(t\right)d\hat{\omega } \end{array}$
Pros	Effective capability at capturing uncertainty; flexible in modeling.	Effective in high-dimensional spaces, can generate high-quality samples.	Capture dynamic systems well; useful for time-dependent processes.
Cons	Can be computationally intensive and may require large datasets.	Require careful tuning; may be sensitive to model architecture.	Complexity in solving, may require numerical methods for solutions.

**Table 3:** Research questions of this proposed work.

Ques No.	Questions	Objectives	Category
RQ 1	How effectively do diffusion models perform across various health informatics tasks?	To outline the advantages of the diffusion-based models in medical imaging.	Performance
RQ 2	How comprehensive and diverse are the datasets utilized in diffusion model applications within health informatics?	To explore the medical image dataset diversification where diffusion-based methods are employed and then present a concise taxonomy.	Dataset
RQ 3	To what extent is explainability incorporated into diffusion models for health informatics, and how is it achieved?	To evaluate the performance reliability of the diffusion models and provide transparency.	Explainability
RQ 4	What is the degree of clinician involvement in diffusion model research and implementation in health informatics?	To underscore the clinical importance of the diffusion-based model, whether the model’s outcome is relevant or not.	Clinical Importance
RQ 5	What are the key challenges for the real-time deployment of diffusion models in health informatics systems?	To identify emerging trends, tools, and technologies to shape the future of diffusion methods in health informatics.	Future Research

**Table 4:** Search queries to obtain relevant articles.

Search Query	Connector	Technique
Diffusion AND models or methods or process or application	AND	medical AND imaging or health AND informatics AND image AND generation or segmentation or denoising or translation or reconstruction or classification or super AND resolution or anomaly AND detection

**Table 5:** Anomaly detection utilizing diffusion models. ✓ denotes presence in the study, and × denotes absence in the study.

Reference	Dataset Name	Dataset Availability	Dataset Type	Model	Results	XAI	Clinician Involvement	Real-Time Implementation
[70]	BraTS2021	Public	MRI	Conditional-Guided Diffusion Model	Dice: 85.6, MSE: 0.0071, Precision: 74.8, Specificity: 99.3%	✓	×	×
[71]	PhotoBox	Private	Dermatology	Score-Based Diffusion Model	ROC: 91.2% Dice: 0.358 IoU: 0.231	×	✓	×
[51]	Neurofeedback Skull-Stripped (NFBS)	Public	MRI scan	Multi-scale noise Diffusion	Dice: 0.383 ± 0.258 IoU: 0.269 ± 0.204 AUC: 0.863 ± 0.107	✓	×	×
[72]	BraTS 2021, MSLUB	Public	MRI scan	Patched Diffusion Models	BraTS 2021-(Dice: 49 ± 0.84, AUPRC: 54.07 ± 1.06), MSLUB-(Dice: 10.47 ± 1.27, AUPRC: 10.58 ± 0.85)	×	✓	×
[74]	Medical Segmentation Decathlon Dataset from OUHSC	Private	CT	Denoising Diffusion Implicit Models	Dice Score: 0.123 Accuracy: 92.30%	✓	✓	×
[73]	ADNI	Public	MRI and PET	Conditioned Diffusion Models	SSIM: 0.8913 ± 0.0745 MSE: 0.0028 ± 0.0031 PSNR: 30.5090 ± 2.4281	✓	✓	×
[75]	IXI, ATLAS	Public, On Request	MRI and X-ray	Diffusion Models with Temporal Harmonization for Optimal Restoration	ATLAS (Dice score: 20.41) IXI (Recall: 83.33%, F1 score: 73.76%)	×	✓	×
[76]	MSLUB and BraTS2021	Public	MRI	Masked-Denoising Diffusion Probabilistic Model	MSLUB-(DICE: 10.71 ± 0.62, AUPRC: 10.59 ± 0.57) BraTS2021-(DICE: 53.02 ± 1.34, AUPRC: 59.04 ± 1.26)	×	×	×

**Table 6:** Classification utilizing diffusion models. ✓ denotes presence in the study, and × denotes absence in the study.

Reference	Dataset Name	Dataset Availability	Dataset Type	Model	Results	XAI	Clinician Involvement	Real-Time Implementation
[77]	Cataract (Nuclear Sclerosis) dataset, Knee Osteoarthritis (OA) dataset, Braille dataset, and Diabetic Retinopathy dataset	Public	X-ray, Retinal OCT	Latent Diffusion Model and CNN model	Cataract 97.52% Knee OA 68.66% Diabetes 93.75% Braille 93.50%	×	×	×
[79]	PMG2000 HAM10000 APTOS2019	Public	Ultrasound, Dermatology Retinal OCT	Dual-Granularity Conditional Guidance Diffusion Model	PMG2000 93.1% HAM10000 90.6% APTOS2019 85.8%	×	×	×
[78]	GLySAC and CoNSeP	Public	Histopathology	Semantic Diffusion Model	Diff Mix Accuracy GLySAC: 71.6% CoNSeP: 87.3%	×	×	×
[80]	Atlas	On Request	MRI and CT scan	Synchronized Anisotropic Diffusion Equation	MI: 3.0144 ± 0.4917 $Q^{0}$: 0.5102 ± 0.1496 $Q_{AB/F}$: 0.5581 ± 0.04222 NCIE: 0.8150 ± 0.0030 VIFF: 0.4374 ± 0.0608 FSIM: 0.8050 ± 0.0258 IFC: 4.9106 ± 2.6251 VSI: 0.9175 ± 0.0088	×	×	×
[81]	HAM10000 APTOS	Public	Dermatology Retinal OCT	Accuracy Adaptive Sampling Diffusion Model	HAM10000- Macro F1: 81.97 B-ACC: 81.50 MCC: 80.64 APTOS- Macro F1: 73.02 B-ACC: 72.27 MCC: 80.76	×	×	×
[82]	1. In-house dataset (Breast cancer NPC datasets) 2. AAPM OpenKBP challenge	1. Private 2. Public	X-Ray, Retinal OCT	Distance-Aware Diffusion Model	Breast Cancer (MAE: 1.076 ± 0.232) NPC dataset (MAE: 1.676 ± 0.387) OpenKBP dataset (MAE: 2.382 ± 0.925)	✓	×	×

**Table 7:** Diffusion models in denoising. ✓ denotes presence in the study, and × denotes absence in the study.

Reference	Dataset Name	Dataset Availability	Dataset Type	Model	Results	XAI	Clinician Involvement	Real-Time Implementation
[86]	ISIC, Kvasir, and BUSI	Public	Ultrasound, Dermatology	Feature Map Denoising Diffusion Model	ISIC: (F1: 91.02%, IoU 83.76%), Kvasir: (F1: 92.07%, IoU 85.55%), BUSI: (F1: 81.68%, IoU 69.65%).	×	×	×
[83]	Chest X-Ray Kaggle Lung Dataset IDRiD	Public	X-Ray, CT, Retinal OCT	Generalized Hybrid Denoising Diffusion Model	Frechet Inception Distance: X-ray—88.64%, CT—67.87%, Retinal OCT—1.02	✓	✓	×
[84]	Two Brain PET Image Datasets ([F]FDG, [F]MK-6240)	On Request	PET	Denoising Diffusion Probabilistic Model	[F]FDG, Model Version (DDPM-PET) (SSIM: 0.92, PSNR: 35) [F]MK-6240 1/4 Low-Dose Dataset, Model Version (DDPM-PETMR) (SSIM: 0.91, PSNR: 34 ± 1) FJMK 1/6 Low-Dose Dataset, Model Version (DDPM-MR-PETCon) (SSIM: 0.85, PSNR: 30.5 ± 1)	×	✓	×
[87]	Retinal Image; Brain MRI Image; Lung CT Image; Spider X-Ray Image	Private	Retinal OCT MRI CT X-ray	Variable-Order Fractional Diffusion Model	Retinal Image (Avg. PSNR: 42.75, MSE: 0.44) Brain MRI Image (Avg. PSNR: 43.8, MSE: 0.15) Lung CT Image (Avg. PSNR: 44.36, MSE: 0.19) Spider X-Ray Image (Avg. PSNR: 44.04, MSE: 0.24)	×	×	×
[85]	Custom PET Image Dataset	Private	PET	PET-Consistent Denoising Diffusion Probabilistic Model	Normalized MAE: 1.278 ± 0.122% PSNR: 33.783 ± 0.824 SSIM: 0.964 ± 0.009	×	✓	×
[88]	Optic Nerve Head (ONH)	Private	Retinal OCT	Unsupervised Diffusion Probabilistic Model	SNR: 40.94 ± 1.78 PSNR: 74.67 ± 0.58 CNR: 2.12 ± 0.71	×	×	×

**Table 8:** Diffusion models in generation. ✓ denotes presence in the study, and × denotes absence in the study.

Reference	Dataset Name	Dataset Availability	Dataset Type	Model	Results	XAI	Clinician Involvement	Real-Time Implementation
[89]	MRI Super-Resolution, Chest X-Ray and MRI Inpainting	Public and On Request	MRI, X-Ray	Conditional Denoising Diffusion Probabilistic Models	LPIPS: 2.96 FID: 0.582	×	✓	×
[90]	Clinical Stereotactic Radiosurgery Dataset BraTS2021	On Request and Public	MRI	Med-Denoising Diffusion Probabilistic Models	Dice score: 0.6675 ± 0.2623 IoU: 0.5495 ± 0.2489 Recall: 0.6673 ± 0.2949	×	✓	×
[21]	Messidor, ISIC, PneumoniaMnist, BreastMnist	Public	Retinal OCT, Ultrasound, and X-Ray	Class-Conditional Diffusion Model	Messidor: (FID: 17.47, IS: 1.39), ISIC: (FID: 159.29, IS: 4.27), PneumoniaMnist: (FID: 124.59, IS: 2.80), BreastMnist: (FID: 206.25, IS: 2.17)	×	✓	×
[93]	Lung Cohorts, NCK-CRC, and PatchCamelyon	Private and Public	Histopathology	Infinite Diffusion–Hierarchical Diffusion Model	Classification results: Lung Cohorts (0.8520 ± 0.007), NCK-CRC (0.847 ± 0.044) and PatchCamelyon (0.641 ± 0.035)	×	✓	×
[94]	Brain MRI (ADNI), Chest CT, Breast MRI, and Knee MRI	Public and Private	MRI and CT	Denoising Diffusion Probabilistic Models	ADNI: 90.0% Chest CT: 86%, Breast MRI: 94%, Knee MRI: 96%	×	✓	×
[91]	AIROGS, CRCDX, CheXpert	Public	Retinal OCT, Histopathology, and X-Ray.	Latent Denoising Diffusion Probabilistic Models	AIROGS—(FID: 11.63, Precision: 0.70.) CRCDX—(FID: 30.03, Precision: 0.66) CheXpert—(FID: 17.28, Precision: 0.68.)	×	✓	×
[92]	Chest X-Ray 14 ACDC, SILVER07, MIMIC-CXR and Openl	Public	CT, X-Ray, MRI	Cross-Guided Diffusion Model	Chest X-ray14: PSNR: 40.16, SSIM: 98.27, NIQE 2.49. ACDC: PSNR: 42.48, SSIM: 98.92, NIQE 2.02. SILVER07: PSNR: 39.51, SSIM: 95.68, NIQE 2.31. MIMIC-CXR: FID: 0.48, NIQE 2.91. Openl: FID: 0.92, NIQE 2.66	×	✓	×
[95]	ACDC	Public	MRI	Deformable Diffusion	PSNR: 30.725, NMSE: 0.466, Dice: 0.802	×	×	×
[96]	BraTS2018, IXI	Public	MRI	Conditioned Latent Diffusion Model	BraTS2018: PSNR—28.26 ± 3.13 SSIM—93.65 ± 3.02 IXI: PSNR—32.24 ± 2.95 SSIM—96.95 ± 2.26	×	×	×

**Table 9:** Diffusion models in reconstruction. ✓ denotes presence in the study, and × denotes absence in the study.

Reference	Dataset Name	Dataset Availability	Dataset Type	Model	Results	XAI	Clinician Involvement	Real-Time Implementation
[24]	NBIA Database	Public	CT image (abdominal, head)	DDDM	(PSNR; SSIM; LPIPS) Abdomen: (38.132; 0.921; 0.0486) Head: (42.066; 0.984; 0.0420)	×	×	×
[97]	(a) IXI Dataset	Public	Brain MRI	AnaDiff	(PSNR; SSIM) | 4× accel. T1: (42.1 ± 1.6; 99.1 ± 0.3) T2: (41.9 ± 1.6; 98.9 ± 0.2) PD: (42.6 ± 1.9; 99.1 ± 0.2)	×	✓	×
	(b) fastMRI				T1: (40.2 ± 1.7; 95.9 ± 1.4) T2: (37.7 ± 0.8; 96.2 ± 0.4) Flair: (36.2 ± 2.6; 92.5 ± 4.8)			
[98]	(a) fastMRI	Public	Brain MRI	SSDiffRecon	(PSNR; SSIM) T1: (40.1; 96.5) T2: (37.7; 96.6) PD (38.4: 99.0) (4× accel., best)	×	✓	×
	(b) IXI Dataset				(PSNR; SSIM) T1: (42.3; 99.3) T2: (45.9; 99.1) Flair: (98.4; 99.0) (4× accel., best)			
[99]	fastMRI	Public	Knee MRI	MC-DDPM	PD—PSNR 36.69 PDFS—PSNR 33.00 PD—SSIM 0.905 PDF—SSIM 0.735 (4× accelerations, best)	✓	✓	×
[103]	fastMRI	Public	Brain MRI	MI-PS	(NRMSE; SSIM) (0.071; 0.894) | 3× accel.	×	×	×
[100]	(a) FastMRI Brain Multi-Coil Dataset	Public	Brain MRI	SMRD	(PSNR; SSIM) (36.5; 0.89) | 4× accel.	×	×	✓
	(b) Mridata Multi-Coil Knee Dataset		Knee MRI		(35.0; 0.84) | 12× accel.			
[101]	(a) SIAT	Private	Brain MRI	CM-DM	(PSNR; SSIM; MSE, e-4) T1-GE: (40.58; 0.9387; 0.901) | 10× accel. T1-weighted: (36.55; 0.8899; 2.215) | 8× accel.	✓	✓	×
	(b) fastMRI+	Public			(36.84; 0.9036; 2.072) | Test-2, 8× accel.			
[102]	fastMRI	Public	Knee MRI	HFS-SDE	(NMSE; PSNR; SSIM) | 10 fold (0.65 ± 0.26; 33.28 ± 1.83; 84.09 ± 4.11)	✓	✓	✓
			Brain MRI		(1.43 ± 0.82; 31.06 ± 0.87; 79.97 ± 5.14)			
[104]	(a) GLIST-RT	Public	Brain MRI	MAR-CDPM	(NMSE; PSNR; MS-SSIM; SSIM; VIF; MS-GMSD) *p* values < 0.05 (statistically significant)	×	✓	×
	(b) MR-ART				(NMSE; PSNR; MS-SSIM; SSIM; VIF; MS-GMSD) *p* values < 0.05 (statistically significant)			

**Table 11:** Diffusion models in super-resolution. ✓ denotes presence in the study, and × denotes absence in the study.

Reference	Dataset Name	Dataset Availability	Dataset Type	Model	Results	XAI	Clinician Involvement	Real-Time Implementation
[117]	(a) IXI Dataset	Public	Brain MRI	ASSRDM	(PSNR; SSIM) (37.53; 0.9863)	×	×	×
	(b) fastMRI				(37.66; 0.9877)			
[118]	The Amsterdam Open MRI Collection	Public	Brain MRI	DDPM-based	PSNR: 24.63 SSIM: 0.7847 LPIPS: 0.031	×	✓	×
[119]	IXI Dataset	Public	Brain MRI	InverseSR	(SSIM; PSNR) Decoder: (0.803 ± 0.030; 29.64 ± 1.64) LDM: (0.754 ± 0.038; 27.92 ± 1.60)	×	✓	×
[116]	Baby Connectome Project (BCP)	Public	Brain MRI	Cas-DiffCom	PSNR: 24.15 SSIM: 0.81	×	✓	×
[120]	Harvard Medical School Database	Public	Brain MRI	TFS-Dif	(MSE; VIF; SSIM; PSNR; LPIPS; MAE; RMSE; AG) (2021.23; 0.577; 0.818; 15.27; 0.319; 44.80; 103.64; 7.529) | 2× accel.	✓	×	×
[121]	(a) FFHQ	Public	Face	ResDiff	(PSNR; SSIM; FID) (26.73; 0.818; 70.54)	×	×	×
	(b) CelebA				(27.16; 0.797; 38.47)			
	(c) Div2k		General		(27.16; 0.797; 38.47)			
	(d) Urban100				(27.43; 0.82; 42.35)			
[115]	Local Dataset	Private	Fadaveric Femurs Micro-CT	DDPM-based	GSS: Pearson r > 0.99	×	✓	✓
[124]	AOMIC brain MRI	Public	Brain MRI	3D-SRDM	PSNR: 21.560 ± 2.669 SSIM: 0.605 ± 0.111 LPIPS: 0.109 ± 0.080	×	×	×
[122]	(a) FastMRI	Public	Knee MRI	DiffMSR	(PSNR; SSIM) (33.78; 0.8765)	×	×	×
	(b) Clinical Brain	unknown	Brain MRI		(34.12; 0.9362)			
	(c) Clinical Tumor				(33.85; 0.9274)			
	(d) Clinical Pelvic		Pelvic MRI		(35.47; 0.9607)			
[123]	fastMRI	Public	Knee MRI	Score-based reversed diffusion	PSNR: 31.13 SSIM: 0.908 LPIPS: 0.218 mAP: 0.314	✓	✓	×

**Table 12:** Diffusion models in translation. ✓ denotes presence in the study, and × denotes absence in the study.

Reference	Dataset Name	Dataset Availability	Dataset Type	Model	Results	XAI	Clinician Involvement	Real-Time Implementation
[127]	SynthRAD2023	Public	Brain MRI and CT	FDDM	FID: 29.8828 SSIM: 0.8255 ± 0.0544 PSNR: 36.4282 ± 1.7404	×	✓	×
[128]	(a) Lumbar Spine (L)	Private	MRI and CT scans	Contour Diff	(Dice; ASSD) (0.683; 1.432)	×	✓	×
	(b) Hip and Thigh (H&T)				(0.731; 3.139)			
	(c) SPIDER Lumbar Spine (L-SPIDER)	Public	MRI scans		(0.633; 2.066)			
[125]	(a) Posterior–Anterior DE Chest Radiographs	Public	X-ray	CMDM	(PSNR; SSIM; MAE) Soft-tissue: (44.27 ± 2.33; 0.991 ± 0.002; 0.369 ± 0.041) Bone: (44.58 ± 3.16; 0.992 ± 0.003; 0.348 ± 0.038)	×	✓	✓
	(b) In-House Brain MRI	Private	Brain MRI		T1→T2: (27.93 ± 1.54; 0.898 ± 0.042; 0.202 ± 0.051) T1→Flair: (27.98 ± 1.54; 0.901 ± 0.044; 0.201 ± 0.051)			
	(c) AAPM Low-Dose CT Grand Challenge	Public	Whole-body CT		1/6 Sparse: (46.42 ± 1.22; 0.986 ± 0.003; 0.302 ± 0.039) 1/4 Sparse: (47.02 ± 1.25; 0.990 ± 0.003; 0.299 ± 0.038)			
[126]	MRSpineSeg Challenge	Public	Spine CT	DDIM	(L1; MSE; PSNR; SSIM; VIFp) T1-weighted: (0.0131; 0.0020; 27.89; 0.887; 0.411) T2-weighted: (0.0131; 0.0021; 27.36; 0.898; 0.401)	×	✓	×
[131]	(a) The Gold Atlas	Public	Pelvic MRI and CT	MIDiffusion	CSLMI:10.16	×	✓	✓
	(b) CuRIOUS		Brain MRI		CSLMI:3.62			
	(c) IXI Dataset				CSLMI:6.32			
[130]	(a) MRI-CT Brain	Public	Brain MRI and CT	TGDM	PSNR: 18.62 SSIM: 0.612 FID: 71.03	×	✓	×
	(b) Prostate-MRI-US-Biopsy		Prostate MRI and ultrasound		FID: 143.33			
[129]	(a) Head and Neck Dataset	Public	CT and CBCT	FGDM	(PSNR; SSIM; FID) with source (27.8; 0.908; 24.0)	×	✓	×
	(b) Lung Dataset				(28.4; 0.906; 42.4)			
	(c) Organs at Risk (OARs) Dataset				(24.0; 0.857; 94.8)			
	(d) Head MR T1 Dataset		Brain MRI		(21.2; 0.805; 59.3)			
[23]	(a) IXI Dataset	Public	Brain MRI	SynDiff	(PSNR; SSIM) T2→ T1: (30.42 ± 1.40; 94.77 ± 1.26) T1→T2: (30.32 ± 1.46; 94.28 ± 1.32) PD→T1: (30.09 ± 1.36; 94.99 ± 1.17) T1→PD: (30.85 ± 1.56; 94.03 ± 1.12) PD→T2: (33.64 ± 0.86; 96.58 ± 0.36) T2→PD: (35.47 ± 1.15; 96.98 ± 0.36)	×	×	×
	(b) BraTS Benchmark				T2→ T1: (28.90 ± 0.73; 93.79 ± 0.95) T1→T2: (27.10 ± 1.26; 92.35 ± 1.27) Flair→T1: (26.47 ± 0.69; 89.37 ± 1.50) T1→Flair: (26.45 ± 1.02; 87.79 ± 1.67) Flair→T2: (26.75 ± 1.18; 91.69 ± 1.50) T2→Flair: (28.17 ± 0.90; 90.44 ± 1.48)			
	(c) Pelvic MRI-CT Dataset		Pelvic MRI and CT		accel. T2→CT: (26.71 ± 0.63; 87.32 ± 2.84) accel. T1→CT: (25.47 ± 1.09; 85.00 ± 2.10)			
[132]	(a) BraTS2021	Public	Brain MRI	MS-SPADE	(PSNR; SSIM; SSIM) T1→T2: (25.818 ± 0.857; 0.079 ± 0.016; 0.904 ± 0.012) T2→Flair: (25.074 ± 1.085; 0.098 ± 0.021; 0.867 ± 0.018)	×	×	×
	(b) IXI Dataset				T1→PD: (27.729 ± 0.885; 0.068 ± 0.022; 0.921 ± 0.018)			

**Table 13:** Future research directions.

FRQ	Issues/Topics	Future Research Path
FRQ1	Inference time and memory load	Implementing model pruning techniques to reduce the number of parameters and computational complexity. Utilizing quantization methods to decrease memory footprint and speed up inference.
FRQ2	Artifacts in sparse-view CT and extreme under-sampling in MRI; image quality optimization	Applying advanced reconstruction algorithms such as iterative methods or deep learning-based techniques to improve image quality. Using synthetic data generation and augmentation to enhance training robustness against under-sampling. Incorporating regularization techniques to minimize artifacts in the reconstructed images. Implementing multi-scale approaches to handle varying levels of data sparsity and quality.
FRQ3	Handling diverse imaging conditions and motion types; reducing retraining needs	Developing adaptive models with real-time motion correction capabilities using domain adaptation techniques. Implementing modular model architectures that can be easily updated with new modules for different conditions. Exploring transfer learning and pre-trained networks to handle new imaging conditions without extensive retraining.
FRQ4	Utilization of labeled and unlabeled data in diffusion models; semi-supervised learning optimization	Designing semi-supervised learning algorithms that combine diffusion models with self-training or pseudo-labeling techniques. Developing methods for dynamic data selection to prioritize informative unlabeled samples. Exploring hybrid loss functions that balance supervised and unsupervised objectives.
FRQ5	Validation process for clinical applicability; role of medical professionals	Involving authors from medical science domain for throughout supervision to ensure the study is applicable to clinical practices.
FRQ6	Auxiliary tasks and multi-scale feature aggregation in segmentation models	Utilizing multi-scale feature extraction methods such as dilated convolutions or pyramidal networks to capture varying levels of detail. Applying feature aggregation techniques like attention mechanisms or feature fusion layers to improve model performance. Conducting ablation studies to evaluate the impact of different auxiliary tasks and aggregation strategies.
FRQ7	Feature grouping strategies; handling varying feature correlations and importance	Developing dynamic feature grouping algorithms that adapt based on input data characteristics. Implementing feature selection techniques like LASSO or principal component analysis (PCA) to manage feature importance. Exploring correlation-based feature weighting methods to adjust the significance of features during training.
FRQ8	Balancing image quality and computational efficiency in 3D super-resolution	Applying image super-resolution techniques such as residual networks or generative adversarial networks (GANs) to improve quality while optimizing efficiency. Utilizing computational optimization techniques such as multi-resolution training or patch-based processing to balance quality and speed.
FRQ9	Maintaining/enhancing performance without pre-processing steps	Developing end-to-end training pipelines that integrate data preprocessing within the model architecture. Exploring methods for adaptive preprocessing, such as automated data enhancement techniques.
FRQ10	Building on one-to-many modality translations	Developing multi-modal translation frameworks that can handle complex interactions between various imaging modalities. Implementing domain adaptation techniques to ensure accurate translations across different modalities.
FRQ11	Convergence of Diffusion-Based Models	Developing hybrid architecture combining diffusion and foundation models, using large cross-domain datasets to improve generalization across imaging modalities. Investigating parameter-efficient fine-tuning (e.g., LoRA and adapters) to adapt large diffusion backbones to label-scarce clinical tasks.
FRQ12	Integration of federated learning, physics-guided, and state-space methods	Integrating physics-informed methods into diffusion sampling to improve clinical accuracy. Developing federated diffusion frameworks for privacy-preserving, multi-center hospital training. Combining diffusion models with state-space architectures (e.g., Mamba and autoregressive hybrid models) to improve scalability and long-range coherence.

## Data Availability

No new data were created or analyzed in this study. Data sharing is not applicable to this article.

## Supplementary Materials

The following supporting information can be downloaded at https://www.mdpi.com/article/10.3390/diagnostics16020211/s1, Reference [67] is cited in the supplementary materials.
